# Dickkopf Proteins and Their Role in Cancer: A Family of Wnt Antagonists with a Dual Role

**DOI:** 10.3390/ph14080810

**Published:** 2021-08-18

**Authors:** Irina Giralt, Gabriel Gallo-Oller, Natalia Navarro, Patricia Zarzosa, Guillem Pons, Ainara Magdaleno, Miguel F. Segura, José Sánchez de Toledo, Lucas Moreno, Soledad Gallego, Josep Roma

**Affiliations:** 1 Laboratory of Translational Research in Child and Adolescent Cancer, Vall d’Hebron Research Institute, Hospital Universitari Vall d’Hebron, Universitat Autònoma de Barcelona, 08035 Barcelona, Spain; irina.giralt@vhir.org (I.G.); gabriel.gallo@vhir.org (G.G.-O.); natalia.navarro@vhir.org (N.N.); patricia.zarzosa@vhir.org (P.Z.); guillem.pons@vhir.org (G.P.); ainara.magdaleno@vhir.org (A.M.); miguel.segura@vhir.org (M.F.S.); jossanchez@vhebron.net (J.S.d.T.); lucas.moreno@vhebron.net (L.M.); sgallego@vhebron.net (S.G.); 2 Pediatric Oncology and Hematology Department, Hospital Universitari Vall d’Hebron, Universitat Autònoma de Barcelona, 08035 Barcelona, Spain

**Keywords:** Wnt signaling, Wnt antagonists, Dickkopf, DKK

## Abstract

The Wnt signaling pathway regulates crucial aspects such as cell fate determination, cell polarity and organogenesis during embryonic development. Wnt pathway deregulation is a hallmark of several cancers such as lung, gastric and liver cancer, and has been reported to be altered in others. Despite the general agreement reached by the scientific community on the oncogenic potential of the central components of the pathway, the role of the antagonist proteins remains less clear. Deregulation of the pathway may be caused by overexpression or downregulation of a wide range of antagonist proteins. Although there is growing information related to function and regulation of Dickkopf (DKK) proteins, their pharmacological potential as cancer therapeutics still has not been fully developed. This review provides an update on the role of DKK proteins in cancer and possible potential as therapeutic targets for the treatment of cancer; available compounds in pre-clinical or clinical trials are also reviewed.

## 1. Overview of the Wnt Pathway

The Wnt signaling pathway is a highly conserved pathway involved in many processes of embryonic development such as proliferation, differentiation and epithelial-mesenchymal transition in a wide range of tissues. During the early stages of embryogenesis, the Wnt pathway plays a crucial role in body–axis formation. Later, this pathway is needed for the development of many organs such as brain, kidney, reproductive tract and mammary glands, among others [1]. The pathway can be activated in a canonical or non-canonical manner.

The canonical pathway is β-catenin-dependent and requires the binding of Wnt ligands to receptors [2]. The first step involves the Wnt ligands, Frizzled (Fz) receptors and the low-density lipoprotein receptor-related protein 5/6 (LRP5/6). Wnt ligands are secreted and bind to Fz. LRP5/6 co-receptors are necessary for mediating Wnt signaling. In an “ON” state (Figure 2A), the interaction Wnt-Fz-LRP5/6 activates the disheveled protein (Dsh) (in humans encoded by *DVL1* gene), which plays a role as a key switch for Wnt signaling. Dsh constitutes an element which, depending on the context, can activate different downstream effectors and modify the response. The main function of Dsh is to inhibit the β-catenin destruction complex. This complex comprises the interaction of adenomatosis polyposis coli (APC), glycogen synthase kinase 3 (GSK3), casein kinase 1 alpha (CK1α) and axin. The inactive complex permits the accumulation of β-catenin in cytoplasm to further translocate it to the nucleus. Nuclear β-catenin interacts with T-cell factor/lymphoid enhancer factors (TCF/LEF, transcription factors), and the co-activators CREB-binding protein (CBP) and P300. As a result, the transcription of Wnt target genes is activated [2,3]. Target genes include c-Myc, cyclin D1, axin2, CD44 and c-Jun, among others [4,5,6]. In an “OFF” state (Figure 2B), there is no binding of Wnt ligands and the destruction complex is active. In this state, CK1α and GSK3 phosphorylate β-catenin, thereby producing its ubiquitination and degradation by E3 ubiquitin ligases [2].

The non-canonical Wnt pathway is β-catenin-independent and can activate additional pathways. Although the non-canonical pathway is under study and not yet well understood, it can be classified in two groups: the planar cell polarity pathway and the Wnt/Ca^2+^ pathway (for a general overview of the non-canonical Wnt, see [7], for Wnt/planar cell polarity [8] and for Wnt/Ca^2+^ pathway [7]). Activation of the non-canonical pathways also involves the binding of Wnt ligands to the Fz receptor, but independently of the LRP5/6 co-receptor. The non-canonical pathway highlights the complexity and tight regulation of Wnt signaling, providing a large number of receptor/ligand combinations and several layers of regulation, which render this pathway highly context-specific and underline its participation in different cellular responses and diseases [2,3].

It is clear that Wnt signaling plays a pivotal role in developmental and cellular processes such as cell proliferation, migration and fate determination. However, its deregulation has been identified as a key mechanism in different diseases including cancer. Several aberrant regulatory processes such as mutations, overexpression and downregulation mechanisms have been described for members of the Wnt signaling [9]. Among the proteins involved in these mechanisms, some have been described as antagonists of the Wnt pathway. They are usually classified in different families/groups (Table 1) and are able to modulate Wnt signaling at 3 levels: Wnt ligands, LRP proteins and/or Fz receptors. Herein, we will focus on the Dickkopf (DKK) family with emphasis on cancer, and current inhibitors/activators under development for this particular Wnt antagonist family will be also reviewed.

## 2. The Dickkopf (DKK) Family and Its Role in Cancer

DKK is a family of soluble (mainly extracellular) LRP5/6 antagonists that prevent the formation of the Fz-LRP6 complex. In the absence of DKKs, the Wnt ligands form a ternary complex with Fz and Lrp5/6, which promotes stabilization of β-catenin, thereby activating the pathway. However, the binding of DKK to Kremen (a family of two transmembrane proteins characterized by their kringle domain) enable the formation of a three-component complex with LRP5/6, which leads to rapid endocytosis and removal of this protein from the plasma membrane. The inhibitory function of DKK proteins depends on the presence of the appropriate Kremen proteins [10].

**Figure 1:**
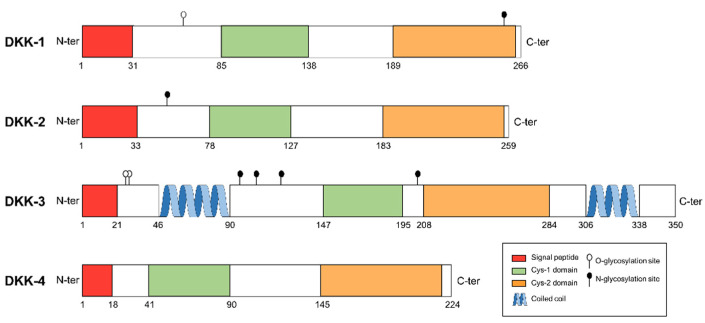
Domain structure of human DKK protein family. Cysteine-rich domain 1 (Cys1) and Cys2 domains are shared by the four members of DKK family. Signal peptide, coiled-coil region and glycosylation sites are also shown.

In humans, four *DKK* genes have been reported: *DKK-1*, *DKK-2*, *DKK-3* and *DKK-4*, with *DKK-1* being the most studied and characterized [11,12]. DKK proteins show little sequence similarity (Figure 1).

Only two cysteine-rich domains are highly preserved among all the family members: (1) the N-terminal cysteine-rich domain (formerly called Cys1) that is unique for the members of DKK family; and (2) the C-terminal cysteine-rich domain (or Cys2) which has a 10-cysteine pattern. The Cys2 domain is responsible for LRP6 binding and further Wnt inhibition, whereas the Cys1 domain modulates this interaction. Nevertheless, this described function for Cys1 appears to be functional only in DKK-1 [12]. The antagonistic effect of DKK family on LRP5/6 is specific to the Wnt canonical pathway. However, it has been described that DKK-1 could activate the non-canonical Wnt/planar cell polarity [13].

It is not easy to attribute a pro-oncogenic or tumor suppressor function to each antagonist in the context of cancer given that the majority may play a dual role and act either by promoting or repressing cancer in a context-dependent manner. The ability of DKK proteins to inhibit the Wnt pathway suggests a function for them as possible tumor suppressor genes owing to the most preponderant pro-oncogenic role of overactivation of the pathway. However, in some tumors where the Wnt pathway has anti-oncogenic functions (i.e., induction of differentiation), DKK proteins may also act as oncogenes. A low expression of *DKK* genes, due to either methylation of the promoters or other mechanisms, has been described in colorectal cancer, kidney cancer, hepatocellular carcinoma and gastric cancer, among many others [14,15,16,17]. In contrast, the overexpression of *DKK* constitutes a valuable indicator of prognosis and chemoresistance and can even improve diagnostic accuracy in some tumors [18,19,20,21]. Thus, the function of tumor suppressors or oncogenes is context-dependent and, in some cases, both functions have been described for the same tumor type, thereby increasing the complexity of reaching an easily understandable description. Although the precise role of each DKK is not fully elucidated, the implications in cancer are evident and their dual role in tumors is related to the complexity of the Wnt pathway. In the following sections, more details are provided for each particular type of DKK together with information related to clinical application, current DKK inhibitors and ongoing clinical trials.

### 2.1. Dickkopf-1 (DKK-1)

DKK-1 is by far the most studied member of the family. It was first identified in *Xenopus laevis* as a secreted protein able to inhibit the Wnt pathway and with an essential role in head formation during development [22]. Its expression has also been described in paraxial mesoderm and vertebra formation and, along with Sonic Hedgehog, in the formation and maintenance of the apical ectodermal crest, a critical component of limb formation [23].

DKK-1 is a potent antagonist of the Wnt pathway able to bind the LRP6 receptor with high affinity. Two models have been described for the DKK-1 molecular mechanism of action. In one, DKK-1 performs its function by competing with Wnt ligands to bind the LRP6 receptor. In the second, owing to the ability of DKK-1 to bind to Kremen proteins, a ternary complex (Kremen-LRP6-DKK) is formed and rapidly internalized and degraded, thereby reducing the presence of LRP receptors in the cell membrane and, in turn, promoting inactivation of the pathway. In addition to these two β-catenin-dependent mechanisms, DKK-1 can also act in a β-catenin-independent manner, but this mechanism is not well understood and remains under study [24,25,26].

In terms of cancer, the strong duality of the role of DKK-1 is noteworthy. In many tumors, DKK-1 clearly acts only as an oncogene (Supplementary Table S1). For instance, several studies showed a relationship between DKK-1 overexpression and worse prognosis in common tumors such as lung cancer (including non-small cell lung cancer) [27,28] and rare malignancies such as chondrosarcoma [29,30]. However, the setting is highly complex and a clear oncogenic or anti-oncogenic role of DKK-1 in a given tumor type is not always clear, with a plethora of examples in which references to both sides may be found. Interestingly, *DKK-1* expression would play a dual role in childhood cancers. For example, its expression has been found reduced in neuroblastoma, suggesting a tumor-suppressor role [31]; however, its expression in this tumor may help the cells to resist chemotherapy [32]. By contrast, in osteosarcoma, DKK-1 is overexpressed in patient serum and its downregulation inhibited metastasis in a preclinical model [33,34]. Finally, it has also been reported that, in Ewing Sarcoma, DKK-1 constitutes a target of the oncogenic EWSR1-FLI1 chimeric protein, and that DKK-1 inhibition could contribute to progression of tumors of the Ewing family [35].

### 2.2. Dickkopf-2 (DKK-2)

DKK-2 has been reported to be coordinately expressed with other DKK family members during organogenesis in a multitude of organs in several organisms, including humans. Its expression, together with DKK-1 and DKK-3, in different primordial structures such as heart, tooth, kidney, palate and limb buds has been reported [36].

DKK-2 has been described, like the rest of DKK, as a canonical Wnt signaling inhibitor in numerous in vitro and in vivo studies. It shares its main mechanism of action with DKK-1, i.e., binding to LRP6 receptors [37]. Interestingly, DKK-1 and DKK-2 share a 50% identity in their N-terminal domains and 70% in their C-terminal cysteine-rich domains (Figure 1). The similarity with DKK-1 evidences a coordinated antagonistic role during *Xenopus* embryogenesis, where DKK-2 is able to synergize with the Fz receptor family. Therefore, DKK-2 could act as an activator or inhibitor of the Wnt pathway, depending on the cellular context [38].

Regarding a role in cancer and in accordance with its dual performance in Wnt pathway regulation, both functions, as oncogene and tumor suppressor, have been attributed to DKK-2 (Supplementary Table S2). Interestingly, its relationship with tumor immunity evasion in some subsets of melanoma and colorectal tumors, where DKK-2 depletion activates natural killer (NK) cells and CD8+ T lymphocytes and impedes tumor progression, has also been described. The molecular mechanism for immunity evasion involves LRP5 but is independent of LRP6 and Wnt canonical pathway [39]. A recent study that reported a role of DKK-2 in chemoresistance in breast cancer it is also noteworthy, with a description of the long non-coding RNA GAS5 as an endogenous “sponge” competing with miRNA-221-3p to regulate its target DKK-2, which in turn, inhibits the activation of Wnt pathway [40]. Interestingly, DKK-2 has also been involved in the enhancement of stemness in colorectal cancer, via the activation of the tyrosine-kinase Src and degradation of HNF4α1 protein [41]. Despite the abundance of previous preclinical studies in cancer, none have reached clinical phases to date. However, there are also examples of anti-oncogenic function. Thus, it has been recently described that DKK-2 expression, together with G protein-coupled estrogen receptor (GPER) correlate with overall survival, thereby indicating a possible prognostic impact as well as a potential for treatment strategies addressing interactions between estrogen and Wnt signaling in ovarian cancer [42]. The lack of effective inhibitors or activators for DKK-2 can be explained by the difficulties in maintaining the native structure and to obtain the recombinant protein in vitro. To solve these issues, a strategy to stabilize the DKK-2 structure [43], offering the possibility of designing and identifying specific DKK-2 inhibitors, was recently described. In addition, other strategies have been explored: the generation of a long-acting variant of DKK-2 with an increased serum half-life, which permits systemic administration for the modulation of Wnt signaling [44], and the development of a DKK-2 neutralizing monoclonal antibody (called 5F8). The administration of 5F8 has been shown to impair tumor growth and to increase the survival in orthotopic models for Lewis lung carcinoma [45] and colon adenocarcinoma [39]. These recent advances increase the value of DKK-2 as a therapeutic target, opening up the possibility of developing effective inhibitors against this DKK member.

### 2.3. Dickkopf-3 (DKK-3)

Like other DKK, DKK-3 is also involved in embryo development and morphogenesis [46]. However, in contrast to the other DKK proteins, *DKK-3* does not belong to the same paralogous chromosome group, suggesting an early evolutionary divergence of this member [47].

The role of DKK-3 in Wnt signaling inhibition remains unclear since it is not able to interact with Kremen or LRP5/6 receptors [47,48]. Nevertheless, DKK-3 downregulation has been correlated with β-catenin accumulation in several cancers; however, the underlying molecular mechanisms remain to be fully understood [49,50,51].

Of note, the most prominently reported function of DKK-3 in cancer is as a tumor suppressor since it is known to have reduced expression in immortalized cells [52]. In addition, downregulation of DKK-3 has been correlated to tumor cell proliferation in several solid cancers and hematologic malignancies (Supplementary Table S3). Consistent with its role as a tumor suppressor, DKK-3 overexpression (by adenovirus-mediated transduction of an eukaryotic expression vector) represses tumor cell growth by inducing endoplasmic reticulum stress which in turn triggers JNK-dependent apoptosis and overexpression of IL-7 in cancer cells [52,53,54,55]. Moreover, exogenous overexpression of DKK-3 has been shown to induce G0/G1 arrest together with an increase in p21 [56]. In this respect, Ad-REIC/DKK-3 (adenoviral vector encoding the full-length *DKK-3* gene) has been proposed as a non-genotoxic gene-based therapy, alone or in combination with conventional antineoplastic treatments, for several cancers such as prostate, testicular, glioma or breast cancer [53,54,57,58] (see Section 4). The oncogene function of DKK-3, albeit less frequent, has also been identified. Its pro-oncogenic activities have been described particularly in head and neck squamous cell carcinoma. In comparison to normal tissue, the aberrant accumulation of DKK-3 found in squamous cell carcinoma cytoplasm seems to exert oncogenic effects and has been associated with the accumulation of β-catenin [59]. Moreover, like other DKK members, secreted DKK-3 is able to modulate T-cell immune responses, thereby supporting its role as a tumor microenvironment modulator [60]. In this regard, DKK-3 stromal expression has been associated with induction of tumor-promoting cancer-associated fibroblasts (CAF) in breast, colorectal and ovarian cancers [61]. Under physiologic and pathologic conditions, DKK-3 is able to regulate angiogenesis and several studies have reported an increase in the number of blood vessels expressing higher amounts of DKK-3 compared to its normal counterparts in different types of cancer [62,63,64]. Owing to the function in angiogenesis, the combination of future DKK-3 inhibitors together with other angiogenesis inhibitors available could produce a synergistic effect and constitute an effective approach for future therapies.

### 2.4. Dickkopf-4 (DKK-4)

DKK-4 is the least studied member of the family and its role during development or disease has barely been described. However, it has been related to ectodermal appendage morphogenesis, including hair follicles, teeth and mammary gland formation during mouse embryonic development [65]. DKK-4 acts exclusively as an inhibitor of Wnt signaling and is able to interact with the LRP5/6 receptor [66,67]. DKK-4 can also bind to Kremen, although how this interaction may inhibit Wnt activity has not yet been described [68]. Although the underlying molecular mechanism for DKK-4 has not been elucidated to date, evidence indicates its role through non-canonical Wnt signaling [69].

Like the other three family members, the role of DKK-4 has been described as both a tumor suppressor and oncogene in various cancers (Supplementary Table S4). Although DKK-4 is the least studied member, its implication in cancer is sustained by small but growing evidence. Besides the studies that identify its possible role as a tumor suppressor or oncogene, DKK-4 expression has been strongly related to cancer cell migration (Supplementary Table S4). Interestingly, a possible function in mediating drug resistance in colorectal cancer cells has been described [70], and DKK-4 inhibition has been proposed to enhance chemosensitivity to current treatments in colorectal [71] and non-small cell lung cancer [72]. Owing to its role in chemosensitivity, DKK-4 inhibition would improve current therapies in cases where chemoresistance is observed.

## 3. DKK Proteins as Diagnostic and/or Prognostic Factor in Cancer

Detecting DKK levels may improve the diagnosis and prediction of patient outcome. In fact, this approach has been explored in the last decade for several tumors. However, results are often contradictory, even for one given tumor type. In addition, the dual role of DKK proteins in cancer makes their utility as diagnostic/prognosis factor less attractive. For example, in hepatocellular carcinoma, only one study found decreased DKK-1 levels at mRNA and protein levels together with high levels of the methylation promoter for this gene in human samples [16]. Since more evidence in hepatocellular carcinoma supports an oncogenic function, could opposing evidence be considered? After reviewing the current knowledge on the DKK family, our answer is yes, albeit with considerations.

The study of DKK in clinical practice will need to define the ‘what, when, where and how’ for information-gathering. The levels of mRNA, protein, methylation and even single nucleotide polymorphism (SNP) have been analyzed for almost all DKK (what). However, discrepancies were found. For example, after mRNA and protein levels of DKK-3 were analyzed in endometrial adenocarcinoma and control samples, significant differences were found only at protein level. Interestingly, only mRNA expression correlated with tumor grade [73]. This latter finding highlights the complexity of the different molecular mechanisms regulating DKK expression and the diverse functions it may exert in different parts of the body and even in the same disease. The selection of ‘what’ probably depends on ‘when’ detection occurs. Most studies included samples usually obtained at the time of biopsy or diagnosis. However, some studies focused on the potential of DKK analysis over time in the same patient. As an example, a DKK-1 expression increase could be an early phenomenon during multiple myeloma relapse [74], indicating that levels of this protein and their clinical significance can vary according to tumor progression (when) [75,76].

Furthermore, the wide variety of samples used could explain the controversy regarding the function reported in a given tumor. Samples range from tumor tissues (fresh or fixed) to serum and other liquid biopsies such as urine, seminal plasma or bone marrow according to tumor location. One example of how DKK levels can vary according to the type of sample analyzed (where) is the case of DKK-3 in prostate cancer (Supplementary Table S3). Generally, prostate tumors display low *DKK-3* expression possibly due to high promoter methylation [77,78]. However, one study revealed high DKK-3 protein levels in seminal plasma of prostate cancer patients [79]. The authors attributed this inconsistency to the expression of DKK-3 in tumor neo-vasculature, which highlighted the difficulties in defining the ideal sample to be analyzed.

In recent decades, advances in molecular techniques have led to significant improvements in methods and analyses (how). Recent meta-analyses validated the role of DKK-1 and its use as diagnostic and prognostic factors in hepatocellular carcinoma and gastric cancer [80,81,82,83,84]. A similar analysis was recently reported for DKK-3 through Oncomine, TCGA and Kaplan-Meier plotter databases in different tumors [85]. One interesting approach is to consider *DKK* genes as an expression signature (DKK genes alone or in combination with other genes) to enhance accuracy of their use as predictors of clinical outcome and develop new therapeutic strategies [86,87,88]. This methodology has been particularly successful in ovarian cancer, where the use of a signature expression of 7 genes (including *DKK-3*) was able to segregate patients according to their longer or shorter survival [86].

## 4. DKK Family as Emerging Molecular Targets for Anti-Cancer Drugs

**Figure 2:**
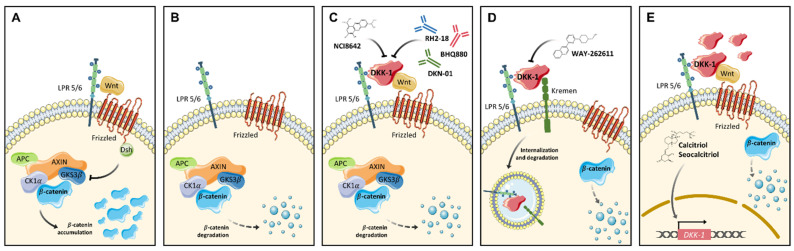
Strategies to target DKK proteins. (**A**) When Wnt signaling is active, Wnt ligands bind to Fz receptor and the LRP5/6 co-receptor, thereby producing the inhibition of the degradation complex (APC-Axin-CK1α-GKS3β) and consequent β-catenin accumulation and stabilization. (**B**) If Wnt ligands do not bind, the destruction complex is active and the degradation of β-catenin is produced. (**C**) DKK-1 blocks Wnt and LPR5/6 interaction by activating the degradation complex and further inhibition of the signaling by β-catenin degradation. NCI8642, RH2-18, BHQ880 and DKN-01 are able to block this specific DKK-1 activity. (**D**) The interaction of DKK-1 with LPR5/6 and Kremen proteins produces DKK-1 internalization together with the LPR5/6 receptor. The WAY-262611 compound can inhibit Kremen-LRP5/6-Dkk-1 complex formation. (**E**) Activators of DKK-1 such as vitamin D analogs induce the transcription of *DKK-1* gene thereby increasing DKK-1 protein levels and the subsequent Wnt signaling inhibition.

The rationale for the identification of possible molecular targets involves either activation or inhibition of the pathway in a context and tumor type dependent manner. In the case of DKK, three different approaches have been proposed: (1) inhibiting DKK proteins, (2) blocking binding with the corresponding receptor to activate Wnt signaling, and (3) increasing *DKK* gene expression through activators (Table 2 and Figure 2). Given the important role of Wnt antagonists in cancer and other diseases, DKK inhibitors stand out among the strategies under development.

### 4.1. Small Molecules

The first reported small molecule with the ability to inhibit DKK-1 was (1-(4-(Naphthalen-2-yl)pyrimidin-2-yl)piperidin-4-yl)methanamine, also known as WAY-262611. This compound facilitates Wnt3a-LRP5 interaction by inhibiting DKK-1, and blocks the formation of the DKK-1-LRP5-Kremen complex (Figure 2D), thereby preventing the internalization of LRP5 receptors and activating the Wnt pathway. Pelletier et al. described, for the first time, the effects of the compound on inhibiting DKK-1 and the promotion of Wnt pathway activation, with additional effects on bone formation [89]. Later, other studies corroborated the effects of the compound on DKK-1 and further activation of Wnt signaling [96]. WAY-262611 has been tested in vitro as a possible therapeutic tool for the treatment of osteolytic bone disease, the most common symptom in multiple myeloma [97], and for rheumatoid arthritis [98]. In vivo, this compound has shown effectiveness in protection against Leishmania infection in mice [99]. Although the therapeutic potential of this small molecule in cancer remains unclear, some findings support its application. For instance, in the work of Choe et al. [98], treatment with WAY-262611 was able to inhibit cell migration and the expression of FAK (focal adhesion kinase), a protein involved in the regulation of invasion and metastasis in many tumors [100].

Another DKK inhibitor is the small molecule NCI8642 (also named IIIC3 or Gallocyanin) which is able to block the interaction between DKK-1 [90,101] and DKK-2 [102] with LRP6 (Figure 2C). Despite the confirmed ability of this compound to inhibit DKK-1-LRP6 interaction and the reported therapeutic potential of the drug in Alzheimer’s disease [103], studies in human cancer are lacking. Therefore, the therapeutic potential of NCI8642 in cancer, albeit strongly plausible, remains to be established.

In recent years, a compound extracted from *Securinega suffruticosa* leaves (used in traditional Chinese medicine) has been shown to inhibit *DKK-1* expression through increased methylation of its promoter. This compound, the L-securinine, reduced the proliferation of lung cancer cells through *DKK-1* inhibition [91]. These examples show the potential of Wnt antagonist proteins as molecular targets for cancer treatment and the efforts to identify new pharmacologic inhibitors of these proteins.

### 4.2. Monoclonal Antibodies

The use of monoclonal antibodies to block DKK proteins constitutes a strategy that has shown promising effects in vitro and in vivo in several tumors [104,105] and other diseases [106]. The rationale for the use of monoclonal antibodies is the putative highly-specific inhibition of the target (Figure 2C). This is the case of the monoclonal antibody RH2-18 that recognizes DKK-1 with the further activation of the Wnt pathway. RH2-18 has been evaluated as a possible treatment for osteoporosis [106]. Other monoclonal antibodies against DKK-1 have been evaluated for the treatment of different tumors. One example is BHQ880 which has been reported to inhibit both growth and metastasis of osteosarcoma in patient derived xenografts mouse models [34]. The application of DKK-1 inhibition by monoclonal antibodies has been analyzed in the treatment of the characteristic osteolytic lesion produced by multiple myeloma progression [107]. Furthermore, BHQ880 has yielded promising results as a therapeutic, by reducing the osteolytic process in multiple myeloma models [105,108].

The promising results observed with RH2-18 and BHQ880 have led to the development and pre-clinical evaluation of other monoclonal antibodies. For instance, the humanized monoclonal therapeutic antibody called DKN-01 inhibits DKK-1 with high affinity and selectivity [109] and has been evaluated for the treatment of ovarian cancer [110]. In addition, the 5F8 antibody was able to specifically inhibit DKK-2 in an in vivo model of colorectal cancer [39]. Furthermore, this inhibition revealed a pivotal role for DKK-2 in modulating the immune response in vivo through activation of NK and CD8+ cells. This last finding opens up the application of DKK as molecular targets in immunotherapy.

### 4.3. Gene and Immunotherapy

The reduced expression in immortalized cell (REIC) gene was originally identified in 2000 and further analysis confirmed that REIC sequence was consistent with the human DKK-3 gene [111]. In recent years, the possible use of the adenoviral vector with the human REIC/DKK-3 (Ad-REIC) as a gene therapy for cancer patients has been described [112]. The method takes advantage of the adenovirus as a vector for the tumor suppressor gene *DKK-3* and induces its expression to increase apoptosis and further tumor growth reduction. However, since this therapy should be locally administered in the tumor and needs imaging guidance, only solid tumors can be treated. Despite this limitation, Ad-REIC treatment constitutes a possible strategy for some tumors such as hepatocellular carcinoma or pancreatic cancer [112,113]. In addition, other studies have tested the potential of Ad-REIC at pre-clinical level in other malignancies using animal models. In these studies, the administration of Ad-REIC produced an impairment of tumor growth in prostate carcinoma, testicular cancer, breast carcinoma, mesothelioma and gastric carcinoma models [114].

Owing to the possible role of DKK in the immune system modulation, different approaches with DKK have also shown promising results and are still under study, especially in multiple myeloma. The potential use of DKK-1 as an antigenic target for immunotherapy is not new. For instance, a work identified DKK-1 as a potent tumor-associated antigen. The authors described the use of DKK-1 peptides to generate specific cytotoxic T lymphocytes that can act specifically and effectively lyse myeloma cells in vitro [115]. However, it is only in recent years that this approach has been analyzed. Active DKK-1 vaccination has been characterized in vitro [116] and in vivo [117], and the improvement through the addition of antigens [118] or peptide sequences [119] has been evaluated for multiple myeloma, thereby increasing the potential therapeutic value of DKK-1 for this tumor.

### 4.4. Activators of Wnt Antagonist Proteins

Antagonist-mediated Wnt inhibition constitutes a hallmark in some tumors, indicating that the function of these genes is to act as tumor suppressors in these diseases. In this context, restoring expression of the Wnt antagonist genes may constitute a further therapeutic approach (Figure 2E). According to this rationale, some molecules can induce their expression. Aguilera et al. affirmed that the induction of *DKK-1* by calcitriol (1alpha,25-dihydroxyvitamin D3), the active form of vitamin D, is able to promote the differentiation of colon cancer cells [95]. Similarly, seocalcitol (EB1089), a vitamin D analog, has been shown to induce the expression of *DKK-1* in colon cancer and pancreatic cancer [95,120], thereby demonstrating its therapeutic potential for cancer treatment.

## 5. Clinical Trials for Wnt Antagonist Proteins in Cancer

A significant number of clinical trials aimed at evaluating the Wnt antagonists’ inhibitors as therapeutic targets are ongoing and involve several of the molecules previously described. However, most of them are still in early phases and only a few have reached phase III (Table 3). As previously mentioned, neutralizing antibodies have shown the most promising results, and lead in the number of clinical trials. One of the most studied antibodies, DKN-01, is currently in phase I for use as a monotherapy or in combination with other chemotherapeutic agents for the treatment of esophagogastric malignancies. Although the detection of some adverse events (cough, leukopenia, neutropenia, anemia and nausea among others), the preliminary results of the study showed good tolerability of DKN-01 and the combination did not alter the patient safety profile [109]. Similarly, the BHQ880 antibody displayed good tolerability alone and in combination with zoledronic acid (a drug used for the treatment of the typical osteolytic lesions produced by multiple myeloma). In addition, the combination evidence a potential clinical activity for the treatment of bone disease in multiple myeloma [121]. Although the tolerance of DKN-01 and BHQ880 is good, the possible benefits need to be confirmed with further studies.

The induction of DKK-3 expression using the adenoviral vector (Ad-REIC) is currently being tested in clinical trials (Table 3). This strategy has been evaluated for the treatment of prostate cancer also with positive results. As example, in a phase I/IIa clinical study with 18 patients with adenocarcinoma of the prostate, the intra-tumoral administration of Ad-REIC prolonged recurrence-free survival, with tumor degeneration and a significant number of TIL (tumor-infiltrating lymphocytes) being observed in the targeted areas of the tumor [124]. In addition, Ad-REIC was evaluated for the treatment of gliomas [55] where further modifications of the system proved to enhance its treatment effectiveness [125], which led to its evaluation in an ongoing phase I/IIa clinical trial [122]. In liver cancer there is also an ongoing phase I/Ib clinical trial [123].

The evaluation of vitamin D and its analogous molecules in cancer treatment merits a special mention in this section. Vitamin D administration for the treatment of cancer patients is a clear example of drug repurposing. Compared to “de novo” drug development, repurposing a drug previously described for other diseases reduces costs and time in the application of a new treatment in clinical practice [126]. The use of vitamin D has been tested for different types of tumor and is the compound related to the DKK family with the highest number of clinical trials (Table 3). Interestingly, all ongoing clinical trials are evaluating the administration of vitamin D together with other chemotherapeutics as a co-treatment or adjuvant therapy. Despite the fact that undesired side effects of using vitamin D supplementation are fewer, it is a point to be considered [127] owing to the characteristics of target patients. In this respect, three clinical trials assessed the possible toxicity of vitamin D and/or side effects in metastatic or recurrent cancer (NCT01588522), in non-small cell lung cancer (NCT00794547) and in hepatocellular carcinoma (NCT00794547) patients. The use of vitamin D to improve cancer treatment has been evaluated, even when its effect on DKK-1 was not identified [128], based on epidemiologic studies that correlate the incidence of cancer and sun exposure [129,130]. Based on this evidence, results vary widely and the benefit of vitamin D remains unclear [131]. However, elucidation of the molecular mechanism involved (for a more detailed description of the vitamin D molecular mechanism and of DKK we recommended the review by Pendás-Franco et al. [132]) would help to improve current therapies with the addition of vitamin D, especially for tumors in which DKK-1 plays a tumor suppressor role.

The number of clinical trials conducted to evaluate DKK inhibitors as therapeutic agents together with other chemotherapeutics has increased. The majority of studies validated DKK inhibition as monotherapy in vitro and in vivo. Although it seems that the DKK inhibition could be effective only for some tumors (e.g., prostate and multiple myeloma), its evaluation together with current therapies would shine the spotlight on DKK as real emerging molecular targets in a wider range of cancers.

## 6. Final Remarks

Wnt antagonist proteins have been related to increased metastatic potential and it has also been demonstrated that they could be used as biomarkers for diagnosis and/or prognosis. Thus, the role of these proteins requires further study to better understand its implication in cancer and also to develop therapies and/or new biomarkers for use in the treatment or diagnosis of cancer. The growing body of information on the role of Wnt antagonist proteins in cancer highlighted their potential as therapeutic targets, but also call attention to the high complexity of a system with an often ambiguous role, and therefore, the difficulty in implementing possible therapies based on these proteins if their role in each particular disease has not been previously characterized. However, their therapeutic potential is confirmed by the development of inhibitors and activators of these Wnt regulators that are currently evaluated in clinical trials. Evidence to date supports a potential use of inhibitor/activator molecules and therapies for cancer treatment, especially in combination with current chemotherapeutic agents, the cornerstone of cancer therapy in the last decade. However, the knowledge gathered to date on Wnt antagonist proteins revealed a marked dual oncogenic/suppressor role which is strongly context-dependent. Therefore, multiple layers of information should be unraveled prior to the establishment of a role for each Wnt antagonist on each particular cancer. Thus, future Wnt antagonist-based cancer therapies must take into account all the information—often not yet available—of what markers should be considered on each case (e.g., RNA, protein or others), in which stages the therapy can be effective, the tumor or metastasis localization (and tissue of origin) and how should be studied (some recent advances in molecular techniques have led to significant improvements in methods such as genomic meta-analyses, expression signatures, among others). This kind of approach may enhance accuracy of DKK as predictors of clinical outcome or even permit the development of new therapeutic strategies.

To our knowledge, this is the first review to focus on the preclinical evidence of the four members of the DKK family. We hope the present review provides better understanding of current knowledge on Wnt antagonist proteins and their therapeutic potential in cancer and highlights the need for additional studies to shed light on the roles played by each antagonist in each particular cancer, which are not yet well defined.

## Figures and Tables

**Table 1:** Antagonist proteins of the Wnt signaling pathway.

Name	Human Gene(s)	Protein(s) Inhibited	Main Cellular Location
WIF1	*WIF1*	Wnt	Extracellular
Cerberus	*CER1*	Wnt	Extracellular
Tiki family	*TIKI (1–2)*	Wnt	Transmembrane
sFRP family	*sFRP (1–5)*	Wnt and Fz	Extracellular
APCDD1	*APCDD1*	Wnt and LRP	Transmembrane
DKK family	*DKK (1–4)*	LRP	Extracellular
SOST/Sclerostin	*SOST*	LRP	Extracellular
Wise	*SOSTDC1*	LRP	Extracellular
MESD	*MESD*	LRP	Endoplasmic reticulum
Waif	*TPBG*	LRP	Transmembrane
IGFBP-4	*IGFBP4*	LRP and Fz	Extracellular
Shisa family	*SHISA (2–9)*	Fz	Transmembrane

**Table 2:** Compounds targeting DKK proteins.

Function	Chemical Structure	Compound Name	Target	Mechanism of Action	Disease (Pre-Clinical)
inhibitor	small molecules	WAY-262611	DKK-1	Inhibits DKK-1 impeding DKK1-LRP5/6 interaction [89]	Rheumatoid arthritis Leishmaniosis AML MM
		NCI8642	DKK-1 and DKK-2	Binds to LRP6 disrupting DKK1-LRP6 interaction [90]	Alzheimer
		L-securinine	DKK-1	Promotes *DKK-1* promoter methylation inhibiting *DKK-1* expression [91]	Lung cancer
	monoclonal antibodies	RH2-18	DKK-1	Binds to DKK-1 Cys-2 domain [92]	Osteoporosis
		BHQ880	DKK-1	Binds to DKK-1 Cys-2 domain [93]	Osteosarcoma MM
		DKN-01	DKK-1	Binds to DKK-1 Cys-2 domain [94]	Solid tumors
		5F8	DKK-2	Blocks the binding of DKK2 to LRP5 [39]	colorectal cancer
activator	vitamin D3	Calcitriol	DKK-1	Induce the transcription of *DKK-1* gene [95]	Osteoporosis Solid tumors
	vitamin D3 analogue	Seocalcitol	DKK-1	Induce the transcription of *DKK-1* gene [95]	Hepatocellular carcinoma

AML: Acute myeloid leukemia, MM: Multiple myeloma.

**Table 3:** Status of clinical trials targeting DKK proteins in cancer ^1^.

Compound/Strategy	Target	Phase	Tumor	ID
DKN-01	DKK-1	I	Esophagogastric Malignancies	NCT02013154
			MM, solid tumors, NSCLC	NCT01457417
			Relapsed MM	NCT01711671
			Intra- or extra-Hepatic biliary system	NCT02375880
		Ib/IIa	Prostate	NCT03837353
		I/II	Hepatocellular Carcinoma	NCT03645980
		II	Endometrial, uterine and ovarian	NCT03395080
			Endometrial and Ovarian	NCT03395080
			BTC and EGC	NCT03818997
BHQ880	DKK-1	I/II	Relapsed MM	NCT00741377
		II	MM	NCT01337752
			Smouldering MM	NCT01302886
Calcitriol (DN-101)	DKK-1	I	Solid tumors	NCT01588522
			CNS	NCT00008086
			Prostate	NCT00004928
			Prostate	NCT00010231
		I/II	Carcinoma, NSCLS	NCT00066885
			NSCLC	NCT00794547
			Metastatic Melanoma	NCT00301067
		II	CCA	NCT01039181
			CRPC	NCT03261336
			AIPC and NSCLC	NCT00285675
			Prostate	NCT00004043
			Prostate	NCT00524589
			Prostate (stage IV)	NCT00017576
			Prostate (metastatic)	NCT00182741
			Pancreas	NCT00238199
			Pancreas	NCT00536770
			Adenocarcinoma	NCT00084864
			BCC	NCT01358045
		II/III	Prostate cancer	NCT00043576
Seocalcitol	DKK-1	III	Hepatocellular Carcinoma	NCT00051532
Dendritic cell vaccine	DKK-1	Early I	Stable and Smouldering MM	NCT03591614
Ad-REIC	DKK-3	I	Prostate	NCT01197209
		I/IIa	Prostate	NCT01931046
		I/IIa	Glioma	[122]
		I/Ib	Liver	[123]
		II	Mesothelioma	NCT04013334

MM: multiple myeloma, NSCLC: Non-Small Cell Lung Cancer, BTC: Biliary tract cancer, EGC: Esophagogastric cancer, CRPC: Castration-resistant prostate cancer, CCA: Cholangiocarcinoma, AIPC: Androgen Independent Prostate Cancer, CNS: Central Nervous System, BCC: Basal cell carcinoma. ^1^ Information from www.clinicaltrials.gov (last access: 26 February 2021).

## Data Availability

Data sharing not applicable.

## Supplementary Materials

The following are available online at https://www.mdpi.com/article/10.3390/ph14080810/s1, Supplementary Table S1: DKK1 deregulation reported in different tumors and its therapeutic potential, Supplementary Table S2: DKK2 deregulation reported in different tumors and its therapeutic potential, Supplementary Table S3: DKK3 deregulation reported in different tumors and its therapeutic potential, Supplementary Table S4: DKK4 deregulation reported in different tumors and its therapeutic potential.
