# Supplementary material for: Dickkopf Proteins and Their Role in Cancer: A Family of Wnt Antagonists with a Dual Role
Source: Pharmaceuticals (Basel). 2021 Aug 18;14(8):810. doi: 10.3390/ph14080810 (PMC8400703; doi:10.3390/ph14080810)
Supplement: Supplementary file 1 [file pharmaceuticals-14-00810-s001.zip › Supplementary Table S1 review DKKs.pdf]

Table S1. DKK1 deregulation reported in different tumors and its therapeutic potential.

DKK-1			
**Tumor**	**Reported function**	**Selected evidence for therapeutic potential**	**References**
**Bladder cancer**	Oncogene	Diagnostic factor	1
		Prognostic marker	2
**Cervical cancer**	Oncogene	Diagnostic factor	3–5
		Prognostic factor	3,4
**Cholangiocarcinoma**	Oncogene	Prognostic factor	6–8
		In vitro proliferation	8
		In vitro migration/invasion	7,8
		In vivo tumor growth	6,8
**Chondrosarcoma**	Oncogene	Diagnostic marker	9
		Prognostic factor	10,11
**Esophageal carcinoma**	Oncogene	Diagnostic factor	12–16
		Prognostic factor	17,18
		In vitro proliferation	15,19
		In vivo tumor growth	19
**Lung cancer**	Oncogene	Diagnostic marker	20–25
		Prognostic factor	5,18,31,21–23,26–30
		In vitro proliferation	5,29,32,33
		In vitro migration/invasion	5,20,29,33,34
		In vivo tumor growth	5,29,32
**Multiple myeloma**	Oncogene	Diagnostic marker	35–37
		Prognostic factor	35,36,38–44
		In vitro proliferation	42,45
		In vivo tumor growth	40,44,46–49
**Osteosarcoma**	Oncogene	Diagnostic marker	50
		Prognostic factor	50
		In vivo tumor growth	51
**Ovarian cancer**	Oncogene	Diagnostic marker	3
		Prognostic factor	3,52,53
		In vivo tumor growth	52
**Pancreatic cancer**	Oncogene	Diagnostic marker	5,54–56
		Prognostic factor	31,54,57
		In vitro migration/invasion	55
**Prostate cancer**	Oncogene	Diagnostic marker	58–60
		Prognostic factor	58,60
		In vivo tumor growth	59,61
**Urothelial carcinoma**	Oncogene	Prognostic factor	62
**Leukemia**	Tumor suppressor	Diagnostic marker	63
		Prognostic factor	64,65
**Medulloblastoma**	Tumor suppressor	*In vitro* proliferation	66
**Melanoma**	Tumor suppressor	Diagnostic marker	67
		In vitro migration/invasion	68
		In vivo tumor growth	69
**Mesothelioma**	Tumor suppressor	*In vitro* proliferation	70
**Neuroblastoma**	Tumor suppressor	Prognostic factor	71
		In vitro proliferation	71,72
		In vitro migration/invasion	73
		In vivo tumor growth	73

**Renal cell Carcinoma/ Wilms tumor**	Tumor suppressor	Diagnostic marker	74
		Prognostic factor	74–76
		In vitro proliferation	75
		In vitro migration/invasion	75
		In vivo tumor growth	75
**Thyroid cancer**	Tumor suppressor	Prognostic factor	77
		In vitro migration/invasion	78
		In vivo tumor growth	79
**Breast cancer**	Oncogene	Diagnostic factor	5,80–83
		Prognostic factor	81–89
		In vitro proliferation	83
		In vivo tumor growth	83,84,90
	Tumor suppressor	*In vitro* proliferation	83,91–93
		In vitro migration/invasion	94
		In vivo tumor growth	90,92
**Colorectal cancer**	Oncogene	Prognostic factor	95–98
	Tumor suppressor	Diagnostic factor	99
		Prognostic factor	100–103
		In vitro proliferation	99,104
		In vitro migration/invasion	101,104
		In vivo tumor growth	100–102
**Endometrial carcinoma**	Oncogene	Diagnostic factor	3
		Prognostic factor	3
	Tumor suppressor	Prognostic factor	105
		In vitro migration/invasion	105,106
**Gastric cancer**	Oncogene	Diagnostic factor	5,107,108
		Prognostic factor	109–113
	Tumor suppressor	Diagnostic factor	114
		Prognostic factor	115
		In vitro proliferation	115
		In vivo tumor growth	115
**Glioma/Glioblastoma**	Oncogene	Prognostic factor	116
	Tumor suppressor	Prognostic factor	117
		In vitro proliferation	118,119
		In vitro migration/invasion	120
**Head and neck carcinoma**	Oncogene	Prognostic factor	121–123
	Tumor suppressor	Prognostic factor	124–126
		In vitro migration/invasion	126
**Hepatocellular carcinoma**	Oncogene	Diagnostic factor	5,127,136–139,128–135
		Prognostic factor	127,132,145–147,135–137,140–144
		In vitro proliferation	133,145
		In vitro migration/invasion	132,133,140,142–145,148
		In vivo tumor growth	142–145
	Tumor suppressor	Diagnostic factor	149
		In vitro proliferation	150,151
		In vitro migration/invasion	150,151
		In vivo tumor growth	150

REFERENCES SUPPLEMENTARY TABLE 1

1. Sun, D. K., Wang, L., Wang, J. M. & Zhang, P. Serum Dickkopf-1 levels as a clinical and prognostic factor in patients with bladder cancer. *Genet. Mol. Res.* **14**, 18181–18187 (2015).

2. Kaba, M. *et al.* Dickkopf-1 levels in Turkish patients with bladder cancer and its association with clinicopathological features. *Asian Pacific J. Cancer Prev.* **15**, 381–384 (2014).

3. Jiang, T., Wang, S., Huang, L. & Zhang, S. Clinical significance of serum DKK-1 in patients with gynecological cancer. *Int. J. Gynecol. Cancer* **19**, 1177–1181 (2009).

4. Jiang, T., Huang, L. & Zhang, S. DKK-1 in serum as a clinical and prognostic factor in patients with cervical cancer. *Int. J. Biol. Markers* **28**, 221–225 (2013).

5. Sato, N. *et al.* Wnt inhibitor Dickkopf-1 as a target for passive cancer immunotherapy. *Cancer Res.* **70**, 5326–5336 (2010).

6. Shi, X. De *et al.* Dickkopf-1 expression is associated with tumorigenity and lymphatic metastasis in human hilar cholangiocarcinoma. *Oncotarget* **7**, 70378–70387 (2016).

7. Shi, R. Y. *et al.* High expression of Dickkopf-related protein 1 is related to lymphatic metastasis and indicates poor prognosis in intrahepatic cholangiocarcinoma patients after surgery. *Cancer* **119**, 993–1003 (2013).

8. Zhang, G. F., Qiu, L., Yang, S. L., Wu, J. C. & Liu, T. J. Wnt/β-catenin signaling as an emerging potential key pharmacological target in cholangiocarcinoma. *Bioscience Reports* (2020) doi:10.1042/BSR20193353.

9. Subramanian, S. *et al.* The gene expression profile of extraskeletal myxoid chondrosarcoma. *Journal of Pathology* vol. 206 433–444 (2005).

10. Chen, C. *et al.* Elevated levels of dickkopf-1 are associated with β-catenin accumulation and poor prognosis in patients with chondrosarcoma. *PLoS One* **9**, e105414 (2014).

11. Zarea, M. *et al.* Diagnostic investigations of DKK-1 and PDCD5 expression levels as independent prognostic markers of human chondrosarcoma. *IUBMB Life* **68**, 597–601 (2016).

12. Peng, Y. H. *et al.* Combined detection of serum Dickkopf-1 and its autoantibodies to diagnose esophageal squamous cell carcinoma. *Cancer Med.* **5**, 1388–1396 (2016).

13. Begenik, H. *et al.* The association between serum Dickkopf-1 levels and esophageal squamous cell carcinoma. *Hum. Exp. Toxicol.* **33**, 785–788 (2014).

14. Darlavoix, T., Seelentag, W., Yan, P., Bachmann, A. & Bosman, F. T. Altered expression of CD44 and DKK1 in the progression of Barrett's esophagus to esophageal adenocarcinoma. *Virchows Arch.* **454**, 629–637 (2009).

15. Li, S. *et al.* Dickkopf-1 is involved in invasive growth of esophageal cancer cells. *J. Mol. Histol.* **42**, 491–498 (2011).

16. Lyros, O. *et al.* Wnt/β-Catenin Signaling Activation beyond Robust Nuclear β-Catenin Accumulation in Nondysplastic Barrett's Esophagus: Regulation via Dickkopf-1. *Neoplasia* **17**, 598–611 (2015).

17. Makino, T. *et al.* Dickkopf-1 expression as a marker for predicting clinical outcome in esophageal squamous cell carcinoma. *Ann. Surg. Oncol.* **16**, 2058–2064 (2009).

18. Yamabuki, T. *et al.* Dikkopf-1 as a novel serologic and prognostic biomarker for lung and esophageal carcinomas. *Cancer Res.* **67**, 2517–2525 (2007).

19. Lu, Z. *et al.* DKK1 maintained cancer stem-like properties of esophageal carcinoma cells via ALDH1A1/SOX2 axis. *Int. J. Clin. Exp. Pathol.* (2017).

20. Li, S. *et al.* Dickkopf-1 is oncogenic and involved in invasive growth in non small cell lung cancer. *PLoS One* **8**, e84944 (2013).

21. Qiao, R. *et al.* Serum dickkopf-1 as a clinical and prognostic factor in nonsmall cell lung cancer patients with bone metastases. *Oncotarget* **8**, 79469–79479 (2017).

22. Shen, L. *et al.* Combined detection of dickkopf-1 subtype classification autoantibodies as biomarkers for the diagnosis and prognosis of non-small cell lung cancer. *Onco. Targets. Ther.* **10**, 3545–3556 (2017).

23. Xiang, X. J. u., Liu, Y. W. e., Chen, D. D. ia. & Yu, S. Differential expression of Dickkopf-1 among non-small cell lung cancer cells. *Mol. Med. Rep.* **12**, 1935–1940 (2015).

24. Yao, X. *et al.* Dickkopf-1 autoantibody is a novel serological biomarker for non-small cell lung cancer. *Biomarkers* **15**, 128–134 (2010).

25. Jiang, X. T. *et al.* Assessing the diagnostic value of serum dickkopf-related protein 1 levels in cancer detection: A case-control study and meta-analysis. *Asian Pacific J. Cancer Prev.* (2014) doi:10.7314/APJCP.2014.15.21.9077.

26. Chu, T., Teng, J., Jiang, L., Zhong, H. & Han, B. Lung cancer-derived Dickkopf1 is associated with bone metastasis and the mechanism involves the inhibition of osteoblast differentiation. *Biochem. Biophys. Res. Commun.* **443**, 962–968 (2014).

27. Dong, L. liang, Qu, L. yun, Chu, L. yan, Zhang, X. hui & Liu, Y. hui. Serum level of DKK-1 and its prognostic potential in non-small cell lung cancer. *Diagn. Pathol.* **9**, 52 (2014).

28. Shi, L. S., Huang, G., Yu, B. & Wen, X. Q. Clinical significance and prognostic value of serum Dickkopf-1 concentrations in patients with lung cancer. *Clin. Chem.* **55**, 1656–1664 (2009).

29. Yao, L. *et al.* Dickkopf-1-promoted vasculogenic mimicry in non-small cell lung cancer is associated with EMT and development of a cancer stem-like cell phenotype. *J. Cell. Mol. Med.* **20**, 1673–1685 (2016).

30. Na, Y., Lee, S. M., Kim, D. S. & Park, J. Y. Promoter methylation of Wnt antagonist DKK1 gene and prognostic value in Korean patients with non-small cell lung cancers. *Cancer Biomarkers* (2012) doi:10.3233/CBM-2012-00295.

31. Kimura, H. *et al.* CKAP4 is a Dickkopf1 receptor and is involved in tumor progression. *J. Clin. Invest.* **126**, 2689–2705 (2016).

32. Pang, H. *et al.* The biological effects of dickkopf1 on small cell lung cancer cells and bone metastasis. *Oncol. Res.* **25**, 35–42 (2017).

33. Kim, I. G. *et al.* Disturbance of DKK1 level is partly involved in survival of lung cancer cells via regulation of ROMO1 and γ-radiation sensitivity. *Biochem. Biophys. Res. Commun.* (2014) doi:10.1016/j.bbrc.2013.11.038.

34. Zhang, J. *et al.* DKK1 promotes migration and invasion of non-small cell lung cancer via β-catenin signaling pathway. *Tumor Biol.* **39**, 1–9 (2017).

35. Politou, M. C. *et al.* Serum concentrations of Dickkopf-1 protein are increased in patients with multiple myeloma and reduced after autologous stem cell transplantation. *Int. J. Cancer* **119**, 1728–1731 (2006).

36. Haaber, J. *et al.* Myeloma cell expression of 10 candidate genes for osteolytic bone disease. Only overexpression of DKK1 correlates with clinical bone involvement at diagnosis. *Br. J. Haematol.* **140**, 25–35 (2008).

37. Hashemi, Z. S. *et al.* Serum DKK1 is correlated with γ peak of serum electrophoresis in multiple myeloma: A multicenter biomarker study. *Biomark. Med.* (2019) doi:10.2217/bmm-2019-0060.

38. Heider, U. *et al.* Serum concentrations of DKK-1 decrease in patients with multiple myeloma responding to anti-myeloma treatment. *Eur. J. Haematol.* **82**, 31–38 (2009).

39. Mabille, C. *et al.* DKK1 and sclerostin are early markers of relapse in multiple myeloma. *Bone* **113**, 114–117 (2018).

40. Yaccoby, S. *et al.* Antibody-based inhibition of DKK1 suppresses tumor-induced bone resorption and multiple myeloma growth in vivo. *Blood* **109**, 2106–2111 (2007).

41. Kaiser, M. *et al.* Serum concentrations of DKK-1 correlate with the extent of bone disease in patients with multiple myeloma. *Eur. J. Haematol.* **80**, 490–494 (2008).

42. Feng, Y., Zhang, Y., Wei, X. & Zhang, Q. Correlations of DKK1 with pathogenesis and prognosis of human multiple myeloma. *Cancer Biomarkers* (2019) doi:10.3233/CBM-181909.

43. Dalla Palma, B. *et al.* Bone marrow Dikkopf-1 levels are a new independent risk factor for progression in patients with smouldering myeloma. *British Journal of Haematology* (2018) doi:10.1111/bjh.15026.

44. Lu, C. *et al.* A novel multi-epitope vaccine from MMSA-1 and DKK1 for multiple myeloma immunotherapy. *Br. J. Haematol.* (2017) doi:10.1111/bjh.14686.

45. Qian, J. *et al.* Dickkopf-1 (DKK1) is a widely expressed and potent tumor-associated antigen in multiple myeloma. *Blood* **110**, 1587–1594 (2007).

46. Heath, D. J. *et al.* Inhibiting dickkopf-1 (Dkkl) removes suppression of bone formation and prevents the development of osteolytic bone disease in multiple myeloma. *J. Bone Miner. Res.* **24**, 425–436 (2009).

47. Fowler, J. A., Mundy, G. R., Lwin, S. T. & Edwards, C. M. Bone marrow stromal cells create a permissive microenvironment for myeloma development: A new stromal role for Wnt inhibitor Dkk1. *Cancer Res.* (2012) doi:10.1158/0008-5472.CAN-11-2067.

48. Park, B. M. *et al.* Cyclized oligopeptide targeting LRP5/6-DKK1 interaction reduces the growth of tumor burden in a multiple myeloma mouse model. *Yonsei Med. J.* (2017) doi:10.3349/ymj.2017.58.3.505.

49. Qian, J. *et al.* Active vaccination with Dickkopf-1 induces protective and therapeutic antitumor immunity in murine multiple myeloma. *Blood* **119**, 161–169 (2012).

50. Lee, N. *et al.* A potential role for Dkk-1 in the pathogenesis of osteosarcoma predicts novel diagnostic and treatment strategies. *Br. J. Cancer* **97**, 1552–1559 (2007).

51. Goldstein, S. D., Trucco, M., Guzman, W. B., Hayashi, M. & Loeb, D. M. A monoclonal antibody against the Wnt signaling inhibitor dickkopf-1 inhibits osteosarcoma metastasis in a preclinical model. *Oncotarget* **7**, 21114–21123 (2016).

52. Wang, S. & Zhang, S. Dickkopf-1 is frequently overexpressed in ovarian serous carcinoma and involved in tumor invasion. *Clin. Exp. Metastasis* **28**, 581–591 (2011).

53. Shizhuo, W., Tao, J., Shulan, Z. & Bing, Z. The expression and significance of Dickkopf-1 in epithelial ovarian carcinoma. *Int. J. Biol. Markers* **24**, 165–170 (2009).

54. Han, S. xia *et al.* Serum dickkopf-1 is a novel serological biomarker for the diagnosis and prognosis of pancreatic cancer. *Oncotarget* **6**, 19907–19917 (2015).

55. Takahashi, N. *et al.* Dickkopf-1 is overexpressed in human pancreatic ductal adenocarcinoma cells and is involved in invasive growth. *Int. J. Cancer* **126**, 1611–1620 (2010).

56. Igbinigie, E., Guo, F., Jiang, S. W., Kelley, C. & Li, J. Dkk1 involvement and its potential as a biomarker in pancreatic ductal adenocarcinoma. *Clinica Chimica Acta* (2019) doi:10.1016/j.cca.2018.11.023.

57. Liu, D. J. *et al.* The role of Dickkopf-1 as a potential prognostic marker in pancreatic ductal adenocarcinoma. *Cell Cycle* **16**, 1622–1629 (2017).

58. Hall, C. L., Daignault, S. D., Shah, R. B., Pienta, K. J. & Keller, E. T. Dickkopf-1 expression increases early in prostate cancer development and decreases during progression from primary tumor to metastasis. *Prostate* **68**, 1396–1404 (2008).

59. Hall, C. L., Bafico, A., Dai, J., Aaronson, S. A. & Keller, E. T. Prostate cancer cells promote osteoblastic bone metastases through Wnts. *Cancer Res.* **65**, 7554–7560 (2005).

60. Rachner, T. D. *et al.* High serum levels of Dickkopf-1 are associated with a poor prognosis in prostate cancer patients. *BMC Cancer* **14**, 649 (2014).

61. Thudi, N. K. *et al.* Dickkopf-1 (DKK-1) stimulated prostate cancer growth and metastasis and inhibited bone formation in osteoblastic bone metastases. *Prostate* (2011) doi:10.1002/pros.21277.

62. Shen, C. H. *et al.* High Dickkopf-1 expression is associated with poor prognosis in patients with advanced urothelial carcinoma. *Exp. Ther. Med.* **1**, 893–898 (2010).

63. Zhou, H. R. *et al.* Relationship between epigenetic changes in Wnt antagonists and acute leukemia. *Oncol. Rep.* (2017) doi:10.3892/or.2017.5509.

64. Suzuki, R. *et al.* Preferential hypermethylation of the Dickkopf-1 promoter in core-binding factor leukaemia. *Br. J. Haematol.* **138**, 624–631 (2007).

65. Pamuk, G. E., Uyanik, M. S., Pamuk, O. N., Maden, M. & Tapan, U. Decreased dickkopf-1 levels in chronic lymphocytic leukemia and increased osteopontin levels in non-hodgkin's lymphoma at initial diagnosis: Could they be playing roles in pathogenesis? *Hematology* **20**, 267–271 (2015).

66. Vibhakar, R. *et al.* Dickkopf-1 is an epigenetically silenced candidate tumor suppressor gene in medulloblastoma1. *Neuro. Oncol.* **9**, 135–144 (2007).

67. Feldmann, R. *et al.* Serum levels of glycoprotein Dickkopf-1 in patients with cutaneous malignant melanoma: A prospective pilot study. *Dermatology* **222**, 171–175 (2011).

68. Chen, J. *et al.* Dickkopf-1 inhibits the invasive activity of melanoma cells. *Clin. Exp. Dermatol.* **37**, 404–410 (2012).

69. Park, H. *et al.* Distinct roles of DKK1 and DKK2 in tumor angiogenesis. *Angiogenesis* **17**, 221–234 (2014).

70. Lee, A. Y. *et al.* Dickkopf-1 antagonizes Wnt signaling independent of β-catenin in human mesothelioma. *Biochem. Biophys. Res. Commun.* **323**, 1246–1250 (2004).

71. Granchi, D. *et al.* Neuroblastoma and bone metastases: Clinical significance and prognostic value of Dickkopf 1 plasma levels. *Bone* **48**, 152–159 (2011).

72. Koppen, A. *et al.* Dickkopf-1 is down-regulated by MYCN and inhibits neuroblastoma cell proliferation. *Cancer Lett.* **256**, 218–228 (2007).

73. Wang, K. P., Bai, Y., Wang, J. & Zhang, J. Z. Morphine protects SH-SY5Y human neuroblastoma cells against Dickkopf1-induced apoptosis. *Mol. Med. Rep.* (2015) doi:10.3892/mmr.2014.2832.

74. Guo, C. C. *et al.* Decreased expression of Dkk1 and Dkk3 in human clear cell renal cell carcinoma. *Mol. Med. Rep.* **9**, 2367–2373 (2014).

75. Hirata, H. *et al.* Wnt antagonist DKK1 acts as a tumor suppressor gene that induces apoptosis and inhibits proliferation in human renal cell carcinoma. *Int. J. Cancer* **128**, 1793–1803 (2011).

76. Wittmann, S. *et al.* New prognostic markers revealed by evaluation of genes correlated with clinical parameters in Wilms tumors. *Genes Chromosom. Cancer* **47**, 386–395 (2008).

77. Zhao, Y. P. *et al.* Downregulation of serum DKK-1 predicts poor prognosis in patients with papillary thyroid cancer. *Genet. Mol. Res.* **14**, 18886–18894 (2015).

78. Cho, S. W. *et al.* Dickkopf-1 inhibits thyroid cancer cell survival and migration through regulation of β-catenin/E-cadherin signaling. *Mol. Cell. Endocrinol.* (2013) doi:10.1016/j.mce.2012.12.007.

79. Cho, S. W. *et al.* Therapeutic potential of Dickkopf-1 in wild-type BRAF papillary thyroid cancer via regulation of β-catenin/E-cadherin signaling. *J. Clin. Endocrinol. Metab.* **99**, E1641–E1649 (2014).

80. Liu, J. T., Guo, W. Bin & Sun, J. Y. Serum Dickkopf-1 acts as a new biomarker in human breast cancer. *Minerva Med.* **108**, 334–340 (2017).

81. Forget, M. A. *et al.* The Wnt pathway regulator DKK1 is preferentially expressed in hormone-resistant breast tumours and in some common cancer types. *Br. J. Cancer* **96**, 646–653 (2007).

82. Sun, W., Shang, J., Zhang, J., Chen, S. & Hao, M. Correlations of DKK1 with incidence and prognosis of breast cancer. *J. B.U.ON.* (2019).

83. Smadja, D. M. *et al.* The Wnt antagonist dickkopf-1 increases endothelial progenitor cell angiogenic potential. *Arterioscler. Thromb. Vasc. Biol.* **30**, 2544–2552 (2010).

84. Voorzanger-Rousselot, N. *et al.* Increased Dickkopf-1 expression in breast cancer bone metastases. *Br. J. Cancer* **97**, 964–970 (2007).

85. Bu, G. *et al.* Breast cancer-derived Dickkopf1 inhibits osteoblast differentiation and osteoprotegerin expression: Implication for breast cancer osteolytic bone metastases. *Int. J. Cancer* **123**, 1034–1042 (2008).

86. Xu, W. H., Liu, Z. Bin, Yang, C., Qin, W. & Shao, Z. M. Expression of Dickkopf-1 and beta-catenin related to the prognosis of breast cancer patients with triple negative phenotype. *PLoS One* **7**, e37624 (2012).

87. Zhou, S. jie, Zhuo, S. rong, Yang, X. qing, Qin, C. xin & Wang, Z. liang. Serum Dickkopf-1 expression level positively correlates with a poor prognosis in breast cancer. *Diagn. Pathol.* **9**, 161 (2014).

88. Kasoha, M. *et al.* Dickkopf-1 (Dkk1) protein expression in breast cancer with special reference to bone metastases. *Clin. Exp. Metastasis* **35**, 763–775 (2018).

89. Rachner, T. D. *et al.* Dickkopf-1 is regulated by the mevalonate pathway in breast cancer. *Breast Cancer Res.* (2014) doi:10.1186/bcr3616.

90. Zhuang, X. *et al.* Differential effects on lung and bone metastasis of breast cancer by Wnt signalling inhibitor DKK1. *Nat. Cell Biol.* (2017) doi:10.1038/ncb3613.

91. Qiao, L., Xu, Z. li, Zhao, T. jun, Ye, L. hong & Zhang, X. dong. Dkk-1 secreted by mesenchymal stem cells inhibits growth of breast cancer cells via depression of Wnt signalling. *Cancer Lett.* **269**, 67–77 (2008).

92. Mikheev, A. M. *et al.* Dickkopf-1 mediated tumor suppression in human breast carcinoma cells. *Breast Cancer Res. Treat.* **112**, 263–273 (2008).

93. Zhou, X. L., Qin, X. R., Zhang, X. D. & Ye, L. H. Downregulation of Dickkopf-1 is responsible for high proliferation of breast cancer cells via losing control of Wnt/B-catenin signaling. *Acta Pharmacol. Sin.* **31**, 202–210 (2010).

94. Niu, J. *et al.* DKK1 inhibits breast cancer cell migration and invasion through suppression of beta-catenin/MMP7 signaling pathway. *Cancer Cell Int.* **19**, 168 (2019).

95. Aguilera, Ó. *et al.* Nuclear DICKKOPF-1 as a biomarker of chemoresistance and poor clinical outcome in colorectal cancer. *Oncotarget* **6**, 5903–17 (2015).

96. Gurluler, E. *et al.* The role of preoperative serum levels for Dickkopf-related protein 1 as a potential marker of tumor invasion in patients with stage II and III colon cancer. *European Review for Medical and Pharmacological Sciences* vol. 18 1742–1747 (2014).

97. Kemik, O. *et al.* Relationship Between Clinicopathologic Variables and Serum and Tissue Levels of Dickkopf-1 in Patients With Rectal Cancer. *J. Investig. Med.* **59**, 947–950 (2011).

98. Sui, Q. *et al.* Dickkopf-related protein 1, a new biomarker for local immune status and poor prognosis among patients with colorectal liver Oligometastases: a retrospective study. *BMC Cancer* **19**, 1210 (2019).

99. Sato, H. *et al.* Frequent epigenetic inactivation of DICKKOPF family genes in human gastrointestinal tumors. *Carcinogenesis* **28**, 2459–66 (2007).

100. Aguilera, O. *et al.* Epigenetic inactivation of the Wnt antagonist DICKKOPF-1 (DKK-1) gene in human colorectal cancer. *Oncogene* **25**, 4116–4121 (2006).

101. Qi, L. *et al.* Dickkopf-1 inhibits epithelial-mesenchymal transition of colon cancer cells and contributes to colon cancer suppression. *Cancer Sci.* **103**, 828–835 (2012).

102. Liu, Z. *et al.* Dickkopf-1 expression is down-regulated during the colorectal adenoma–carcinoma sequence and correlates with reduced microvessel density and VEGF expression. *Histopathology* **67**, 158–166 (2015).

103. Kandimalla, R. *et al.* Methylation of WNT target genes AXIN2 and DKK1 as robust biomarkers for recurrence prediction in stage II colon cancer. *Oncogenesis* **6**, e308–e308 (2017).

104. Maehata, T. *et al.* Transcriptional silencing of Dickkopf gene family by CpG island hypermethylation in human gastrointestinal cancer. *World J. Gastroenterol.* **14**, 2702–2714 (2008).

105. Yi, N., Liao, Q. P., Li, T. & Xiong, Y. Novel expression profiles and invasiveness-related biology function of DKK1 in endometrial carcinoma. *Oncol. Rep.* **21**, 1421–1427 (2009).

106. Yi, N. *et al.* RNA interference-mediated targeting of DKK1 gene expression in Ishikawa endometrial carcinoma cells causes increased tumor cell invasion and migration. *Oncol. Lett.* **6**, 756–762 (2013).

107. Liang, B. *et al.* Serum dickkopf-1 as a biomarker in screening gastrointestinal cancers: A systematic review and meta-analysis. *Onco. Targets. Ther.* (2015) doi:10.2147/OTT.S93152.

108. Gomceli, I. *et al.* A novel screening biomarker in gastric cancer: Serum Dickkopf-1. *Hepatogastroenterology.* **59**, 1661–1664 (2012).

109. Gao, C., Xie, R., Ren, C. & Yang, X. Dickkopf-1 expression is a novel prognostic marker for gastric cancer. *J. Biomed. Biotechnol.* **2012**, 804592 (2012).

110. Lee, H. S. *et al.* Clinical significance of serum and tissue Dickkopf-1 levels in patients with gastric cancer. *Clin. Chim. Acta* **413**, 1753–1760 (2012).

111. Liu, Q. R., Li, Y. F., Deng, Z. Q. & Cao, J. Q. Prognostic Significance of Dickkopf-1 in Gastric Cancer Survival: A Meta-Analysis. *Genet. Test. Mol. Biomarkers* **20**, 170–175 (2016).

112. Hong, S. A. *et al.* Prognostic value of Dickkopf-1 and ß-catenin expression in advanced gastric cancer. *BMC Cancer* **18**, 506 (2018).

113. Zhuang, G. F. *et al.* Expression of serum Dickkopf-1 in gastric cancer patients. *Asian Pac. J. Trop. Med.* **8**, 870–872 (2015).

114. Lu, W. *et al.* DKK1 is epigenetically downregulated by promoter methylation and inhibits bile acid-induced gastric intestinal metaplasia. *Biochem. Biophys. Res. Commun.* (2020) doi:10.1016/j.bbrc.2019.12.109.

115. Jia, X. *et al.* miR-493 mediated DKK1 down-regulation confers proliferation, invasion and chemo-resistance in gastric cancer cells. *Oncotarget* **7**, 7044–7054 (2016).

116. Zhou, Y., Liu, F., Xu, Q. & Wang, X. Analysis of the expression profile of Dickkopf-1 gene in human glioma and the association with tumor malignancy. *J. Exp. Clin. Cancer Res.* **29**, 138 (2010).

117. Götze, S., Wolter, M., Reifenberger, G., Müller, O. & Sievers, S. Frequent promoter hypermethylation of Wnt pathway inhibitor genes in malignant astrocytic gliomas. *Int. J. Cancer* **126**, 2584–2593 (2010).

118. Foltz, G. *et al.* Epigenetic regulation of Wnt pathway antagonists in human glioblastoma multiforme. *Genes and Cancer* **1**, 81–90 (2010).

119. Ma, S. *et al.* Human umbilical cord mesenchymal stem cells inhibit C6 glioma growth via secretion of dickkopf-1 (DKK1). *Mol. Cell. Biochem.* **385**, 277–286 (2014).

120. Guo, K. T. *et al.* The expression of Wnt-inhibitor DKK1 (Dickkopf 1) is determined by intercellular crosstalk and hypoxia in human malignant gliomas. *J. Cancer Res. Clin. Oncol.* **140**, 1261–1270 (2014).

121. Gao, H. *et al.* Elevated DKK1 expression is an independent unfavorable prognostic indicator of survival in head and neck squamous cell carcinoma. *Cancer Manag. Res.* (2018) doi:10.2147/CMAR.S177043.

122. Shi, Y., Gong, H. L., Zhou, L., Tian, J. & Wang, Y. Dickkopf-1 is a novel prognostic biomarker for laryngeal squamous cell carcinoma. *Acta Otolaryngol.* **134**, 753–759 (2014).

123. Katase, N. *et al.* Frequent allelic loss of Dkk-1 locus (10q11.2) is related with low distant metastasis and better prognosis in head and neck squamous cell carcinomas. *Cancer Invest.* **28**, 103–110 (2010).

124. Paluszczak, J. *et al.* Frequent hypermethylation of WNT pathway genes in laryngeal squamous cell carcinomas. *J. Oral Pathol. Med.* **43**, 652–657 (2014).

125. Paluszczak, J. *et al.* The negative regulators of Wnt pathway—DACH1, DKK1, and WIF1 are methylated in oral and oropharyngeal cancer and WIF1 methylation predicts shorter survival. *Tumor Biol.* **36**, 2855–2861 (2015).

126. Ogoshi, K. *et al.* Dickkopf-1 in human oral cancer. *Int. J. Oncol.* **39**, 329–336 (2011).

127. Désert, R. *et al.* “Fibrous nests” in human hepatocellular carcinoma express a Wnt-induced gene signature associated with poor clinical outcome. *Int. J. Biochem. Cell Biol.* **81**, 195–207 (2016).

128. Zhang, J., Zhao, Y. & Yang, Q. Sensitivity and specificity of Dickkopf-1 protein in serum for diagnosing hepatocellular carcinoma: A meta-analysis. *Int. J. Biol. Markers* **29**, e403–e410 (2014).

129. Fouad, Y. M., Mohamed, H. I., Kamal, E. M. & Rasek, M. A. Clinical significance and diagnostic value of serum dickkopf-1 in patients with hepatocellular carcinoma. *Scand. J. Gastroenterol.* **51**, 1133–1137 (2016).

130. Watany, M., Badawi, R., Elkhalawany, W. & Abd-Elsalam, S. Study of dickkopf-1 (DKK-1) gene expression in hepatocellular carcinoma patients. *J. Clin. Diagnostic Res.* **11**, OC32–OC34 (2017).

131. Erdal, H. *et al.* Combination of DKK1 and AFP improves diagnostic accuracy of hepatocellular carcinoma compared with either marker alone. *Turkish J. Gastroenterol.* **27**, 375–381 (2016).

132. Chen, L., Li, M., Li, Q., Wang, C. jie & Xie, S. qiang. DKK1 promotes hepatocellular carcinoma cell migration and invasion through β-catenin/MMP7 signaling pathway. *Mol. Cancer* **12**, 157 (2013).

133. Kim, S. U. *et al.* Serum dickkopf-1 as a biomarker for the diagnosis of hepatocellular carcinoma. *Yonsei Med. J.* **56**, 1296–1306 (2015).

134. Patil, M. A. *et al.* An integrated data analysis approach to characterize genes highly expressed in hepatocellular carcinoma. *Oncogene* **24**, 3737–3747 (2005).

135. Shen, Q. *et al.* Serum DKK1 as a protein biomarker for the diagnosis of hepatocellular carcinoma: A large-scale, multicentre study. *Lancet Oncol.* **13**, 817–826 (2012).

136. Yang, H. *et al.* Dickkopf-1: As a diagnostic and prognostic serum marker for early hepatocellular carcinoma. *Int. J. Biol. Markers* **28**, 286–297 (2013).

137. Younis, Y. S. *et al.* Serum dickopff 1 as a novel biomarker in hepatocellular carcinoma diagnosis and follow up after ablative therapy. *Cancer Manag. Res.* (2019) doi:10.2147/CMAR.S218532.

138. Awad, A. E., Ebrahim, M. A., Eissa, L. A. & El-Shishtawy, M. M. Dickkopf-1 and amphiregulin as novel biomarkers and potential therapeutic targets in hepatocellular carcinoma. *Int. J. Hematol. Stem Cell Res.* (2019) doi:10.18502/ijhoscr.v13i3.1275.

139. Li, Z. *et al.* Diagnostic accuracy of serum dickkopf-1 protein in diagnosis hepatocellular carcinoma: An updated meta-analysis. *Med. (United States)* (2019) doi:10.1097/MD.0000000000016725.

140. Yu, B. *et al.* Elevated expression of DKK1 is associated with cytoplasmic/nuclear β-catenin accumulation and poor prognosis in hepatocellular carcinomas. *J. Hepatol.* **50**, 948–957 (2009).

141. Sakabe, T. *et al.* Expression of cancer stem cell-associated DKK1 mRNA serves as prognostic marker for hepatocellular carcinoma. *Anticancer Res.* **37**, 4881–4888 (2017).

142. Huang, Y. *et al.* Overexpression of Dickkopf-1 predicts poor prognosis for patients with hepatocellular carcinoma after orthotopic liver transplantation by promoting cancer metastasis and recurrence. *Med. Oncol.* **31**, 966 (2014).

143. Tao, Y. M., Liu, Z. & Liu, H. L. Dickkopf-1 (DKK1) promotes invasion and metastasis of hepatocellular carcinoma. *Dig. Liver Dis.* **45**, 251–257 (2013).

144. Tung, E. K. K. *et al.* Clinicopathological and prognostic significance of serum and tissue Dickkopf-1 levels in human hepatocellular carcinoma. *Liver Int.* **31**, 1494–1504 (2011).

145. Zhang, R. *et al.* Dickkopf-1 contributes to hepatocellular carcinoma tumorigenesis by activating the Wnt/beta-catenin signaling pathway. *Signal Transduct. Target. Ther.* **4**, 54 (2019).

146. Wu, X. *et al.* The predictive values of serum dickkopf-1 and circulating tumor cells in evaluating the efficacy of transcatheter arterial chemoembolization treatment on hepatocellular carcinoma. *Medicine (Baltimore).* (2019) doi:10.1097/MD.0000000000016579.

147. Shen, Q. *et al.* High level of serum protein DKK1 predicts poor prognosis for patients with hepatocellular carcinoma after hepatectomy. *Hepatic Oncol.* (2015) doi:10.2217/hep.15.12.

148. Fezza, M., Moussa, M., Aoun, R., Haber, R. & Hilal, G. DKK1 promotes hepatocellular carcinoma inflammation, migration and invasion: Implication of TGF-β1. *PLoS One* (2019) doi:10.1371/journal.pone.0223252.

149. Liang, L. *et al.* Preliminary mechanism on the methylation modification of Dkk-1 and Dkk-3 in hepatocellular carcinoma. *Tumor Biol.* **36**, 1245–1250 (2015).

150. Qin, X. *et al.* Proliferation and migration mediated by Dkk-1/Wnt/β-catenin cascade in a model of hepatocellular carcinoma cells. *Transl. Res.* **150**, 281–294 (2007).

151. Kwack, M. H. *et al.* Analysis of Cellular Changes Resulting from Forced Expression of Dickkopf-1 in Hepatocellular Carcinoma Cells. *Cancer Res. Treat.* **39**, 30 (2007).
