# Supplementary material for: Dickkopf Proteins and Their Role in Cancer: A Family of Wnt Antagonists with a Dual Role
Source: Pharmaceuticals (Basel). 2021 Aug 18;14(8):810. doi: 10.3390/ph14080810 (PMC8400703; doi:10.3390/ph14080810)
Supplement: Supplementary file 1 [file pharmaceuticals-14-00810-s001.zip › Supplementary Table S2 review DKKs.pdf]

Tumor	Reported function	Selected evidence for therapeutic potential	References
**Esophageal carcinoma**	Oncogene	Prognostic factor	1
**Ewing sarcoma**	Oncogene	Prognostic factor	2,3
		In vitro proliferation	3
		In vitro migration/invasion	3
		In vivo tumor growth	3
**Melanoma**	Oncogene	*In vivo* tumor growth	4
**Prostate cancer**	Oncogene	Prognostic factor	5
		In vitro proliferation	5
		In vitro migration/invasion	5
**Breast cancer**	Tumor suppressor	Prognostic factor	6
		In vitro proliferation	6
		In vitro migration/invasion	6
		In vivo tumor growth	6
**Gastric cancer**	Tumor suppressor	Diagnostic factor	7
		Prognostic factor	8,9
		In vitro proliferation	9
		In vitro migration/invasion	9
**Hepatocellular carcinoma**	Tumor suppressor	Prognostic factor	10,11
		In vitro proliferation	11
		In vivo tumor growth	10
**Neuroblastoma**	Tumor suppressor	Prognostic factor	12
**Ovarian cancer**	Tumor suppressor	Prognostic factor	13
		In vitro proliferation	13
		In vitro migration/invasion	13
		In vivo tumor growth	13
**Renal cell carcinoma**	Tumor suppressor	Prognostic factor	14,15
		In vitro proliferation	14
**Colorectal cancer**	Oncogene	Prognostic factor	16
		In vivo tumor growth	16
	Tumor suppressor	Prognostic factor	7,17
		In vitro proliferation	17
		In vitro migration/invasion	17
		In vivo tumor growth	17

Table S2. DKK2 deregulation reported in different tumors and its therapeutic potential.

REFERENCES SUPPLEMENTARY TABLE 2

1. Schiffmann, L. M. *et al.* Dickkopf-2 (DKK2) as context dependent factor in patients with esophageal adenocarcinoma. *Cancers (Basel).* (2020) doi:10.3390/cancers12020451.

2. Staege, M. S. *et al.* DNA microarrays reveal relationship of Ewing family tumors to both endothelial and fetal neural crest-derived cells and define novel targets. *Cancer Res.* (2004) doi:10.1158/0008-5472.CAN-03-4059.

3. Hauer, K. *et al.* DKK2 mediates osteolysis, invasiveness, and metastatic spread in ewing sarcoma. *Cancer Res.* **73**, 967–977 (2013).

4. Park, H. *et al.* Distinct roles of DKK1 and DKK2 in tumor angiogenesis. *Angiogenesis* **17**, 221–234 (2014).

5. Xu, W. *et al.* Dickkopf 2 promotes proliferation and invasion via Wnt signaling in prostate cancer. *Mol. Med. Rep.* **14**, 2283–2288 (2016).

6. Mu, J. *et al.* Dickkopf-related protein 2 induces G0/G1 arrest and apoptosis through suppressing Wnt/β-catenin signaling and is frequently methylated in breast cancer. *Oncotarget* **8**, 39443–39459 (2017).

7. Sato, H. *et al.* Frequent epigenetic inactivation of DICKKOPF family genes in human gastrointestinal tumors. *Carcinogenesis* **28**, 2459–66 (2007).

8. Wang, H. *et al.* Concurrent hypermethylation of SFRP2 and DKK2 activates the Wnt/β-catenin pathway and is associated with poor prognosis in patients with gastric cancer. *Mol. Cells* **40**, 45–53 (2017).

9. Wang, L., Wang, H., Duan, X., Dai, P. & Li, J. Comprehensive Analysis of the Canonical and Non-canonical Wnt Signaling Pathways in Gastric Cancer. *Dig. Dis. Sci.* (2019) doi:10.1007/s10620-019-05606-6.

10. Maass, T. *et al.* Increased liver carcinogenesis and enrichment of stem cell properties in livers of Dickkopf 2 (Dkk2) deleted mice. *Oncotarget* **7**, 28903–28913 (2016).

11. Lin, Y. F. *et al.* Selective Retention of an Inactive Allele of the DKK2 Tumor Suppressor Gene in Hepatocellular Carcinoma. *PLoS Genet.* **12**, e1006051 (2016).

12. Revet, I. *et al.* MSX1 induces the Wnt pathway antagonist genes DKK1, DKK2, DKK3, and SFRP1 in neuroblastoma cells, but does not block Wnt3 and Wnt5A signalling to DVL3. *Cancer Lett.* (2010) doi:10.1016/j.canlet.2009.08.019.

13. Zhu, J., Zhang, S., Gu, L. & Di, W. Epigenetic silencing of DKK2 and Wnt signal pathway components in human ovarian carcinoma. *Carcinogenesis* **33**, 2334–2343 (2012).

14. Hirata, H. *et al.* Wnt antagonist gene DKK2 is epigenetically silenced and inhibits renal cancer progression through apoptotic and cell cycle pathways. *Clin. Cancer Res.* **15**, 5679–5687 (2009).

15. Hirata, H. *et al.* Wnt antagonist gene polymorphisms and renal cancer. *Cancer* (2009) doi:10.1002/cncr.24491.

16. Xiao, Q. *et al.* DKK2 imparts tumor immunity evasion through β-catenin-independent suppression of cytotoxic immune-cell activation. *Nat. Med.* (2018) doi:10.1038/nm.4496.

17. Wang, C. *et al.* Dickkopf-related protein 2 is epigenetically inactivated and suppresses colorectal cancer growth and tumor metastasis by antagonizing Wnt/β-catenin signaling. *Cell. Physiol. Biochem.* **41**, 1709–1724 (2017).
