# Supplementary material for: Dickkopf Proteins and Their Role in Cancer: A Family of Wnt Antagonists with a Dual Role
Source: Pharmaceuticals (Basel). 2021 Aug 18;14(8):810. doi: 10.3390/ph14080810 (PMC8400703; doi:10.3390/ph14080810)
Supplement: Supplementary file 1 [file pharmaceuticals-14-00810-s001.zip › Supplementary Table S3 review DKKs.pdf]

Table S3. DKK3 deregulation reported in different tumors and its therapeutic potential.

Tumor	Reported function	Selected evidence for therapeutic potential	References
**Breast cancer**	Tumor suppressor	Diagnostic factor	[1]
		Prognostic factor	[2–7]
		In vitro proliferation	[3,4,8]
		In vitro migration/invasion	[3,8]
		In vivo tumor growth	[8]
**Gastric cancer**	Tumor suppressor	Prognostic factor	[7,9–13]
		In vitro proliferation	[13]
		In vitro migration/invasion	[13]
**Glioma/Glioblastoma**	Tumor suppressor	Prognostic factor	[14]
		In vitro proliferation	[14–18]
		In vivo tumor growth	[15–18]
**Leukemia**	Tumor suppressor	Prognostic factor	[19]
**Medulloblastoma**	Tumor suppressor	Prognostic factor	[20]
**Neuroblastoma**	Tumor suppressor	Prognostic factor	[21,22]
		In vitro proliferation	[21]
**Osteosarcoma**	Tumor suppressor	*In vitro* proliferation	[23]
		In vitro migration/invasion	[23,24]
		In vivo tumor growth	[23]
**Ovarian cancer**	Tumor suppressor	Prognostic factor	[7,25–27]
		In vitro proliferation	[28]
**Renal cell Carcinoma**	Tumor suppressor	Diagnostic factor	[29,30]
		In vitro proliferation	[31,32]
		In vivo proliferation	[31]
**Thyroid cancer**	Tumor suppressor	Prognostic factor	[33]
**Bladder cancer**	Oncogene	*In vitro* proliferation	[34]
	Tumor suppressor	Diagnostic factor	[35]
		Prognostic factor	[35,36]
		In vitro proliferation	[37–40]
		In vitro migration/invasion	[36]
**Cervical cancer**	Oncogene	Prognostic factor	[25]
	Tumor suppressor	Prognostic factor	[41–44]
		In vitro proliferation	[42,43]
		In vitro migration/invasion	[43]
**Colorectal cancer**	Oncogene	Prognostic factor	[45]
	Tumor suppressor	Diagnostic factor	[46]
		Prognostic factor	[46,47]
		In vitro proliferation	[48]
		In vitro migration/invasion	[48]
**Endometrial carcinoma**	Oncogene	Prognostic factor	[25]
	Tumor suppressor	Prognostic factor	[41,49,50]
		In vitro proliferation	[49]
		In vitro migration/invasion	[49]
		In vivo tumor growth	[49]
**Esophageal carcinoma**	Oncogene	Prognostic factor	[51–53]
		In vitro proliferation	[52]
		In vitro migration/invasion	[51]
		In vivo tumor growth	[51,52]
	Tumor suppressor	Prognostic factor	[54]

**Head and neck carcinoma**	Oncogene	Diagnostic factor	55
		Prognostic factor	56–59
		In vitro proliferation	60,61
		In vitro migration/invasion	60–62
		In vivo tumor growth	60,61
	Tumor suppressor	Diagnostic factor	63,64
**Hepatocellular carcinoma**	Oncogene	Diagnostic factor	65–67
		Prognostic factor	66
		In vitro proliferation	68
		In vivo tumor growth	68
	Tumor suppressor	Diagnostic factor	69,70
		Prognostic factor	70–72
		In vitro proliferation	73
		In vivo tumor growth	73
**Lung cancer**	Oncogene	Prognostic factor	7,74
		In vitro proliferation	74
		In vitro migration/invasion	74
	Tumor suppressor	Prognostic factor	75,76
		In vitro proliferation	76–79
		In vitro migration/invasion	78
		In vivo tumor growth	78,79
**Melanoma/skin cancer**	Oncogene	*In vivo* tumor growth	80
	Tumor suppressor	Prognostic factor	81–83
		In vitro proliferation	82
		In vitro migration/invasion	81,82
		In vivo tumor growth	81,82
**Pancreatic cancer**	Oncogene	Prognostic factor	84
		In vitro proliferation	84
		In vitro migration/invasion	84
		In vivo tumor growth	84
	Tumor suppressor	Diagnostic factor	85,86
		Prognostic factor	87
		In vitro proliferation	85–89
		In vitro migration/invasion	85
		In vivo tumor growth	85,86,88
**Prostate cancer**	Oncogene	Diagnostic factor	90
		In vitro proliferation	91
	Tumor suppressor	Diagnostic factor	92
		Prognostic factor	92–95
		In vitro proliferation	94–99
		In vitro migration/invasion	94,96,100,101
		In vivo tumor growth	98,99,101

REFERENCES SUPPLEMENTARY TABLE 3

1. Kloten, V. *et al.* Promoter hypermethylation of the tumor-suppressor genes ITIH5, DKK3, and RASSF1A as novel biomarkers for blood-based breast cancer screening. *Breast Cancer Res.* (2013) doi:10.1186/bcr3375.

2. Hayashi, T. *et al.* DNA methylation status of REIC/Dkk-3 gene in human malignancies. *J. Cancer Res.*

Clin. Oncol. **138**, 799–809 (2012).

3. Xiang, T. *et al.* Epigenetic silencing of the WNT antagonist Dickkopf 3 disrupts normal Wnt/β-catenin signalling and apoptosis regulation in breast cancer cells. *J. Cell. Mol. Med.* **17**, 1236–1246 (2013).

4. Lorsy, E. *et al.* Loss of Dickkopf 3 promotes the tumorigenesis of basal breast cancer. *PLoS One* **11**, e0160077 (2016).

5. Veeck, J. *et al.* Prognostic relevance of Wnt-inhibitory factor-1 (WIF1) and Dickkopf-3 (DKK3) promoter methylation in human breast cancer. *BMC Cancer* **9**, 217 (2009).

6. Veeck, J. *et al.* Wnt signalling in human breast cancer: Expression of the putative Wnt inhibitor Dickkopf-3 (DKK3) is frequently suppressed by promoter hypermethylation in mammary tumours. *Breast Cancer Res.* **10**, R82 (2008).

7. Chen, G. Y. & Zheng, H. C. The clinicopathological and prognostic significances of Dkk3 expression in cancers: A bioinformatics analysis. *Cancer Biomarkers* (2018) doi:10.3233/CBM-181245.

8. Kawasaki, K. *et al.* REIC/Dkk-3 overexpression downregulates P-glycoprotein in multidrug-resistant MCF7/ADR cells and induces apoptosis in breast cancer. *Cancer Gene Ther.* **16**, 65–72 (2009).

9. Park, J.-M. M. *et al.* Aberrant loss of Dickkopf-3 in gastric cancer: Can it predict lymph node metastasis preoperatively? *World J. Surg.* **39**, 1018–1025 (2015).

10. Yu, J. *et al.* Promoter methylation of the Wnt/β-catenin signaling antagonist Dkk-3 is associated with poor survival in gastric cancer. *Cancer* **115**, 49–60 (2009).

11. Yang, H. J. *et al.* Helicobacter pylori-induced modulation of the promoter methylation of Wnt antagonist genes in gastric carcinogenesis. *Gastric Cancer* (2018) doi:10.1007/s10120-017-0741-6.

12. Kishino, T. *et al.* Integrated analysis of DNA methylation and mutations in esophageal squamous cell carcinoma. *Mol. Carcinog.* **55**, 2077–2088 (2016).

13. Xu, X. Y. *et al.* The roles of REIC gene and its encoding product in gastric carcinoma. *Cell Cycle* (2012) doi:10.4161/cc.19823.

14. Mizobuchi, Y. *et al.* REIC/Dkk-3 induces cell death in human malignant glioma. *Neuro. Oncol.* **10**, 244–253 (2008).

15. Oka, T. *et al.* A super gene expression system enhances the anti-glioma effects of adenovirus-mediated REIC/Dkk-3 gene therapy. *Sci. Rep.* **6**, 33319 (2016).

16. Shimazu, Y. *et al.* Integrin antagonist augments the therapeutic effect of adenovirus-mediated REIC/Dkk-3 gene therapy for malignant glioma. *Gene Ther.* **22**, 146–154 (2015).

17. Fujihara, T. *et al.* Down-regulation of MDR1 by Ad-DKK3 via Akt/NFκB pathways augments the anti-tumor effect of temozolomide in glioblastoma cells and a murine xenograft model. *J. Neurooncol.* (2018) doi:10.1007/s11060-018-2894-5.

18. Hara, K. *et al.* Blocking of the interaction between Wnt proteins and their co-receptors contributes to the anti-tumor effects of adenovirus-mediated DKK3 in glioblastoma. *Cancer Lett.* (2015) doi:10.1016/j.canlet.2014.09.045.

19. Roman-Gomez, J. *et al.* Transcriptional silencing of the Dickkopfs-3 (Dkk-3) gene by CpG hypermethylation in acute lymphoblastic leukaemia. *Br. J. Cancer* **91**, 707–713 (2004).

20. Valdora, F. *et al.* Epigenetic silencing of DKK3 in medulloblastoma. *Int. J. Mol. Sci.* **14**, 7492–7505 (2013).

21. Koppen, A. *et al.* Dickkopf-3 expression is a marker for neuroblasts tumor maturation and is down-regulated by MYCN. *Int. J. Cancer* **122**, 1455–1464 (2008).

22. Revet, I. *et al.* MSX1 induces the Wnt pathway antagonist genes DKK1, DKK2, DKK3, and SFRP1 in neuroblastoma cells, but does not block Wnt3 and Wnt5A signalling to DVL3. *Cancer Lett.* (2010) doi:10.1016/j.canlet.2009.08.019.

23. Lin, C. H. *et al.* Dkk-3, a secreted wnt antagonist, suppresses tumorigenic potential and pulmonary metastasis in osteosarcoma. *Sarcoma* (2013) doi:10.1155/2013/147541.

24. Hoang, B. H. *et al.* Dickkopf 3 Inhibits Invasion and Motility of Saos-2 Osteosarcoma Cells by Modulating the Wnt-β-Catenin Pathway. *Cancer Res.* **64**, 2734–2739 (2004).

25. Jiang, T., Huang, L., Wang, S. & Zhang, S. Clinical significance of serum Dkk-3 in patients with gynecological cancer. *J. Obstet. Gynaecol. Res.* **36**, 769–773 (2010).

26. You, A. *et al.* Expression of the Wnt antagonist DKK3 is frequently suppressed in sporadic epithelial ovarian cancer. *J. Cancer Res. Clin. Oncol.* **137**, 621–627 (2011).

27. Xu, Y. *et al.* Hypermethylation of CDH13, DKK3 and FOXL2 promoters and the expression of EZH2 in ovary granulosa cell tumors. *Mol. Med. Rep.* **14**, 2739–2745 (2016).

28. Takata, A. *et al.* Dkk-3 induces apoptosis through mitochondrial and fas death receptor pathways in human mucinous ovarian cancer cells. *Int. J. Gynecol. Cancer* **25**, 372–379 (2015).

29. Guo, C. C. *et al.* Decreased expression of Dkk1 and Dkk3 in human clear cell renal cell carcinoma. *Mol. Med. Rep.* **9**, 2367–2373 (2014).

30. Kurose, K. *et al.* Decreased expression of REIC/Dkk-3 in human renal clear cell carcinoma. *J. Urol.* **171**, 1314–1318 (2004).

31. Ueno, K. *et al.* Wnt antagonist DICKKOPF-3 (Dkk-3) induces apoptosis in human renal cell carcinoma. *Mol. Carcinog.* **50**, 449–457 (2011).

32. Xu, J. *et al.* Exogenous DKK-3/REIC inhibits Wnt/β-catenin signaling and cell proliferation in human kidney cancer KPK1. *Oncol. Lett.* **14**, 5638–5642 (2017).

33. Yin, D. T. *et al.* DKK3 is a potential tumor suppressor gene in papillary thyroid carcinoma. *Endocr. Relat. Cancer* **20**, 507–514 (2013).

34. Tsujimura, N. *et al.* A novel role of dickkopf-related protein 3 in macropinocytosis in human bladder cancer T24 cells. *Int. J. Mol. Sci.* **17**, 1846 (2016).

35. Urakami, S. *et al.* Combination analysis of hypermethylated Wnt-antagonist family genes as a novel epigenetic biomarker panel for bladder cancer detection. *Clin. Cancer Res.* **12**, 2109–2116 (2006).

36. Ning, J., Yu, W., Cheng, F., Rao, T. & Ruan, Y. MiR-425 Promotes Migration and Invasion in Bladder Cancer by Targeting Dickkopf 3. *J. Cancer* (2020) doi:10.7150/jca.40233.

37. Hirata, T. *et al.* REIC/Dkk-3-encoding adenoviral vector as a potentially effective therapeutic agent for bladder cancer. *Int. J. Oncol.* **41**, 559–564 (2012).

38. Jin, Y. *et al.* Partial sensitization of human bladder cancer cells to a gene-therapeutic adenovirus carrying REIC/Dkk-3 by downregulation of BRPK/PINK1. *Oncol. Rep.* (2012) doi:10.3892/or.2011.1543.

39. Kobayashi, T. *et al.* Mechanistic analysis of resistance to REIC/Dkk-3-induced apoptosis in human bladder cancer cells. *Acta Med. Okayama* **62**, 393–401 (2008).

40. Horikawa, Y. *et al.* Overexpression of REIC/Dkk-3 suppresses the expression of CD147 and inhibits the proliferation of human bladder cancer cells. *Oncol. Lett.* **14**, 3223–3228 (2017).

41. Ryu, S. W. *et al.* Reduced expression of DKK3 is associated with adverse clinical outcomes of uterine cervical squamous cell carcinoma. *Int. J. Gynecol. Cancer* **23**, 134–140 (2013).

42. Lee, E. J. *et al.* Dkk3, downregulated in cervical cancer, functions as a negative regulator of β-catenin. *Int. J. Cancer* **124**, 287–297 (2009).

43. Luo, S. *et al.* MicroRNA-92a promotes cell viability and invasion in cervical cancer via directly targeting Dickkopf-related protein 3. *Exp. Ther. Med.* **14**, 1227–1234 (2017).

44. Kang, W. S. *et al.* Clinico-epigenetic combination including quantitative methylation value of DKK3 augments survival prediction of the patient with cervical cancer. *J. Cancer Res. Clin. Oncol.* (2013) doi:10.1007/s00432-012-1262-7.

45. Zitt, M. *et al.* Dickkopf-3 as a new potential marker for neoangiogenesis in colorectal cancer: expression in cancer tissue and adjacent non-cancerous tissue. *Dis. Markers* **24**, 101–9 (2008).

46. Safari, E., Mosayebi, G. & Khorrami, S. Dkk-3 as a potential biomarker for diagnosis and prognosis of colorectal cancer. *Med. J. Islam. Repub. Iran* (2018) doi:10.14196/mjiri.32.86.

47. Voorham, Q. J. M. *et al.* Promoter methylation of Wnt-antagonists in polypoid and nonpolypoid colorectal adenomas. *BMC Cancer* (2013) doi:10.1186/1471-2407-13-603.

48. Yang, Z. R., Dong, W. G., Lei, X. F., Liu, M. & Liu, Q. S. Overexpression of Dickkopf-3 induces apoptosis through mitochondrial pathway in human colon cancer. *World J. Gastroenterol.* **18**, 1590–1601 (2012).

49. Dellinger, T. H. *et al.* Expression of the Wnt antagonist Dickkopf-3 is associated with prognostic clinicopathologic characteristics and impairs proliferation and invasion in endometrial cancer. *Gynecol. Oncol.* **126**, 259–267 (2012).

50. Eskander, R. N. *et al.* Expression Patterns of the Wnt Pathway Inhibitors Dickkopf3 and Secreted Frizzled-Related Proteins 1 and 4 in Endometrial Endometrioid Adenocarcinoma: An NRG Oncology/Gynecologic Oncology Group Study. in *International Journal of Gynecological Cancer* vol. 26 125–132 (2016).

51. Wang, Z. *et al.* The role of Dickkopf-3 overexpression in esophageal adenocarcinoma. *J. Thorac. Cardiovasc. Surg.* **150**, 377-385.e2 (2015).

52. Kajiwara, C. *et al.* P63-dependent Dickkopf3 expression promotes esophageal cancer cell proliferation via CKAP4. *Cancer Res.* (2018) doi:10.1158/0008-5472.CAN-18-1749.

53. McLaren, P. J. *et al.* Specific gene expression profiles are associated with a pathologic complete response to neoadjuvant therapy in esophageal adenocarcinoma. in *American Journal of Surgery* (2017). doi:10.1016/j.amjsurg.2017.03.024.

54. Liu, J. Bin *et al.* Plasma DNA methylation of Wnt antagonists predicts recurrence of esophageal squamous cell carcinoma. *World J. Gastroenterol.* (2011) doi:10.3748/wjg.v17.i44.4917.

55. Zhang, C. *et al.* Expression pattern of DKK3, dickkopf WNT signaling pathway inhibitor 3, in the malignant progression of oral submucous fibrosis. *Oncol. Rep.* (2017) doi:10.3892/or.2016.5307.

56. Katase, N. *et al.* Deletion at Dickkopf (Dkk)-3 locus (11p15.2) is related with lower lymph node metastasis and better prognosis in head and neck squamous cell carcinomas. *Oncol. Res.* **17**, 273–282 (2008).

57. Katase, N., Nagano, K. & Fujita, S. DKK3 expression and function in head and neck squamous cell carcinoma and other cancers. *Journal of Oral Biosciences* (2020) doi:10.1016/j.job.2020.01.008.

58. Katase, N. *et al.* Absence of Dickkopf (Dkk)-3 protein expression is correlated with longer disease-free survival and lower incidence of metastasis in head and neck squamous cell carcinoma. *Oncol. Lett.* (2012) doi:10.3892/ol.2011.473.

59. Fujii, M. *et al.* Dickkopf (Dkk)-3 and β-catenin expressions increased in the transition from normal oral mucosal to oral squamous cell carcinoma. *J. Mol. Histol.* (2011) doi:10.1007/s10735-011-9357-z.

60. Katase, N. *et al.* DKK3 Overexpression Increases the Malignant Properties of Head and Neck Squamous Cell Carcinoma Cells. *Oncol. Res. Featur. Preclin. Clin. Cancer Ther.* **26**, 45–58 (2017).

61. Katase, N., Nishimatsu, S. I., Yamauchi, A., Yamamura, M. & Fujita, S. DKK3 knockdown confers negative effects on the malignant potency of head and neck squamous cell carcinoma cells via the PI3K/Akt and MAPK signaling pathways. *Int. J. Oncol.* (2019) doi:10.3892/ijo.2018.4667.

62. Katase, N. *et al.* Knockdown of Dkk-3 decreases cancer cell migration and invasion independently of the Wnt pathways in oral squamous cell carcinoma-derived cells. *Oncol. Rep.* **29**, 1349–1355 (2013).

63. Pannone, G. *et al.* WNT pathway in oral cancer: Epigenetic inactivation of WNT-inhibitors. *Oncol. Rep.* **24**, 1035–1041 (2010).

64. Al-Dhohrah, T., Mashrah, M., Yao, Z. & Huang, J. Aberrant DKK3 expression in the oral leukoplakia and oral submucous fibrosis: A comparative immunohistochemical study. *Eur. J. Histochem.* (2016) doi:10.4081/ejh.2016.2629.

65. Erdal, H. *et al.* Combination of DKK1 and AFP improves diagnostic accuracy of hepatocellular carcinoma compared with either marker alone. *Turkish J. Gastroenterol.* **27**, 375–381 (2016).

66. Li, X. *et al.* In-depth analysis of secretome and N-glycosecretome of human hepatocellular carcinoma metastatic cell lines shed light on metastasis correlated proteins. *Oncotarget* (2016) doi:10.18632/oncotarget.8247.

67. Pei, Y. *et al.* Overexpression of Dickkopf 3 in hepatoblastomas and hepatocellular carcinomas. *Virchows Arch.* **454**, 639–646 (2009).

68. Pei, Y. *et al.* GATA4 promotes hepatoblastoma cell proliferation by altering expression of miR125b and DKK3. *Oncotarget* **7**, 77890–77901 (2016).

69. Liang, L. *et al.* Preliminary mechanism on the methylation modification of Dkk-1 and Dkk-3 in hepatocellular carcinoma. *Tumor Biol.* **36**, 1245–1250 (2015).

70. Ding, Z., Qian, Y. Ben, Zhu, L. X. & Xiong, Q. R. Promoter methylation and mRNA expression of DKK-3 and WIF-1 in hepatocellular carcinoma. *World J. Gastroenterol.* **15**, 2595–2601 (2009).

71. Jee, B. A. *et al.* Dynamics of genomic, epigenomic, and transcriptomic aberrations during stepwise hepatocarcinogenesis. *Cancer Res.* (2019) doi:10.1158/0008-5472.CAN-19-0991.

72. Yang, B. *et al.* Methylation of Dickkopf-3 as a prognostic factor in cirrhosis-related hepatocellular carcinoma. *World J. Gastroenterol.* **16**, 755–763 (2010).

73. Hsieh, S. Y., Hsieh, P. S., Chiu, C. T. & Chen, W. Y. Dickkopf-3/REIC functions as a suppressor gene of tumor growth. *Oncogene* **23**, 9183–9189 (2004).

74. Komori, Y. *et al.* Dickkopf-related protein 3 promotes cell adhesion and invasion during progression of lung adenocarcinoma. *Pathol. Int.* (2019) doi:10.1111/pin.12863.

75. Nozaki, I. *et al.* Reduced expression of REIC/Dkk-3 gene in non-small cell lung cancer. *Int. J. Oncol.* **19**, 117–121 (2001).

76. Yue, W. *et al.* Downregulation of Dkk3 activates β-catenin/TCF-4 signaling in lung cancer. *Carcinogenesis* **29**, 84–92 (2008).

77. Tao, L., Huang, G., Chen, Y. & Chen, L. DNA methylation of DKK3 modulates docetaxel chemoresistance in human nonsmall cell lung cancer cell. *Cancer Biother. Radiopharm.* **30**, 100–106 (2015).

78. Wang, Z., Ma, L. J., Kang, Y., Li, X. & Zhang, X. J. Dickkopf-3 (Dkk3) induces apoptosis in cisplatin-resistant lung adenocarcinoma cells via the Wnt/β-catenin pathway. *Oncol. Rep.* **33**, 1097–1106 (2015).

79. Shien, K. *et al.* Anti-cancer effects of REIC/Dkk-3-encoding adenoviral vector for the treatment of non-small cell lung cancer. *PLoS One* **9**, e87900 (2014).

80. Lu, K. H. *et al.* Dickkopf-3 contributes to the regulation of anti-tumor immune responses by mesenchymal stem cells. *Front. Immunol.* (2015) doi:10.3389/fimmu.2015.00645.

81. Kuphal, S. *et al.* Expression of Dickkopf genes is strongly reduced in malignant melanoma. *Oncogene* **25**, 5027–5036 (2006).

82. Shin, J. M. *et al.* The expression pattern and functional role of REIC/Dkk-3 in the development of cutaneous squamous cell carcinoma. *J. Dermatol. Sci.* **84**, 88–96 (2016).

83. Du, G. *et al.* Expression of REIC/Dkk-3 in normal and hyperproliferative epidermis. *Exp. Dermatol.* (2011) doi:10.1111/j.1600-0625.2010.01244.x.

84. Zhou, L. *et al.* Suppression of stromal-derived Dickkopf-3 (DKK3) inhibits tumor progression and prolongs survival in pancreatic ductal adenocarcinoma. *Sci. Transl. Med.* (2018) doi:10.1126/scitranslmed.aat3487.

85. Guo, Q. & Qin, W. DKK3 blocked translocation of β-catenin/EMT induced by hypoxia and improved gemcitabine therapeutic effect in pancreatic cancer Bxpc-3 cell. *J. Cell. Mol. Med.* **19**, 2832–2841 (2015).

86. Uchida, D. *et al.* Potential of adenovirus-mediated REIC/Dkk-3 gene therapy for use in the treatment of pancreatic cancer. *J. Gastroenterol. Hepatol.* **29**, 973–983 (2014).

87. Fong, D. *et al.* Dkk-3 expression in the tumor endothelium: A novel prognostic marker of pancreatic adenocarcinomas. *Cancer Sci.* **100**, 1414–1420 (2009).

88. Sawahara, H. *et al.* Novel REIC/Dkk-3-encoding adenoviral vector as a promising therapeutic agent for pancreatic cancer. *Cancer Gene Ther.* **23**, 278–283 (2016).

89. Gu, Y. M., Ma, Y. H., Zhao, W. G. & Chen, J. Dickkopf3 Overexpression inhibits pancreatic cancer cell growth in vitro. *World J. Gastroenterol.* **17**, 3810–3817 (2011).

90. Zenzmaier, C. *et al.* Elevated levels of Dickkopf-related protein 3 in seminal plasma of prostate cancer patients. *J. Transl. Med.* **9**, 193 (2011).

91. Zenzmaier, C., Sampson, N., Plas, E. & Berger, P. Dickkopf-related protein 3 promotes pathogenic stromal remodeling in benign prostatic hyperplasia and prostate cancer. *Prostate* **73**, 1441–1452 (2013).

92. Kim, M. S., Lee, H. N., Kim, H. J. & Myung, S. C. Single nucleotide polymorphisms in DKK3 gene are associated with prostate cancer risk and progression. *Int. Braz J Urol* (2015) doi:10.1590/S1677-5538.IBJU.2014.0041.

93. Kamińska, K., Białkowska, A., Kowalewski, J., Huang, S. & Lewandowska, M. A. Differential gene methylation patterns in cancerous and non-cancerous cells. *Oncol. Rep.* (2019) doi:10.3892/or.2019.7159.

94. Al Shareef, Z. *et al.* Protective effect of stromal Dickkopf-3 in prostate cancer: opposing roles for TGFBI and ECM-1. *Oncogene* **37**, 5305–5324 (2018).

95. Kawano, Y. *et al.* Regulation of prostate cell growth and morphogenesis by Dickkopf-3. *Oncogene* **25**, 6528–6537 (2006).

96. Kardooni, H. *et al.* CRISPR-mediated reactivation of DKK3 expression attenuates TGF-β signaling in prostate cancer. *Cancers (Basel).* **10**, 165 (2018).

97. Mori, A. *et al.* The downregulation of the expression of CD147 by tumor suppressor REIC/Dkk-3, and its implication in human prostate cancer cell growth inhibition. *Acta Med. Okayama* (2017)

doi:10.18926/AMO/54982.

98. Romero, D. *et al.* Downregulation of Dickkopf-3 disrupts prostate acinar morphogenesis through TGF-β/smad signalling. *J. Cell Sci.* (2013) doi:10.1242/jcs.119388.

99. Chen, J. *et al.* REIC/Dkk-3 stable transfection reduces the malignant phenotype of mouse prostate cancer RM9 cells. *Int. J. Mol. Med.* (2009) doi:10.3892/ijmm_00000293.

100. Romero, D. *et al.* Dickkopf-3 regulates prostate epithelial cell acinar morphogenesis and prostate cancer cell invasion by limiting TGF-β-dependent activation of matrix metalloproteases. *Carcinogenesis* **37**, 18–29 (2016).

101. Edamura, K. *et al.* Adenovirus-mediated REIC/Dkk-3 gene transfer inhibits tumor growth and metastasis in an orthotopic prostate cancer model. *Cancer Gene Ther.* **14**, 765–772 (2007).
