# Supplementary material for: Dickkopf Proteins and Their Role in Cancer: A Family of Wnt Antagonists with a Dual Role
Source: Pharmaceuticals (Basel). 2021 Aug 18;14(8):810. doi: 10.3390/ph14080810 (PMC8400703; doi:10.3390/ph14080810)
Supplement: Supplementary file 1 [file pharmaceuticals-14-00810-s001.zip › Supplementary Table S4 review DKKs.pdf]

Table S4. DKK4 deregulation reported in different tumors and its therapeutic potential.

Tumor	Reported function	Selected evidence for therapeutic potential	References
**Ovarian cancer**	Oncogene	Prognostic factor	1,2
		In vitro migration/invasion	1
**Pancreatic cancer**	Oncogene	Diagnostic factor	3–5
**Renal cell carcinoma**	Oncogene	Diagnostic factor	6,7
		Prognostic factor	7
		In vitro proliferation	6,7
		In vitro migration/invasion	6,7
		In vivo tumor growth	6,7
**Hepatocellular carcinoma**	Tumor suppressor	Diagnostic factor	8
		Prognostic factor	9
		In vitro proliferation	8–12
		In vitro migration/invasion	8,9
		In vivo tumor growth	8–12
**Colorectal cancer**	Oncogene	Diagnostic factor	13
		Prognostic factor	14
		In vitro proliferation	15
		In vitro migration/invasion	15,16
		In vivo tumor growth	17
	Tumor suppressor	*In vitro* proliferation	18
**Gastrointestinal stromal tumor**	Oncogene	Prognostic factor	19
	Tumor suppressor	Diagnostic factor	20
**Lung cancer**	Oncogene	*In vitro* proliferation	21
		In vitro migration/invasion	21
	Tumor suppressor	Prognostic factor	22

REFERENCES SUPPLEMENTARY TABLE 4

1. Wang, S., Wei, H. & Zhang, S. Dickkopf-4 is frequently overexpressed in epithelial ovarian carcinoma and promotes tumor invasion. *BMC Cancer* **17**, 455 (2017).

2. Wang, Y., Lei, L., Chi, Y. G., Liu, L. B. & Yang, B. P. A comprehensive understanding of ovarian carcinoma survival prognosis by novel biomarkers. *Eur. Rev. Med. Pharmacol. Sci.* (2019) doi:10.26355/eurrev_201910_19136.

3. Ouyang, Y., Pan, J., Tai, Q., Ju, J. & Wang, H. Transcriptomic changes associated with DKK4 overexpression in pancreatic cancer cells detected by RNA-Seq. *Tumor Biol.* **37**, 10827–10838 (2016).

4. Park, M. *et al.* Distinct Protein Expression Profiles of Solid-Pseudopapillary Neoplasms of the Pancreas. *J. Proteome Res.* (2015) doi:10.1021/acs.jproteome.5b00423.

5. Zhu, Y. *et al.* Proteomic analysis of solid pseudopapillary tumor of the pancreas reveals dysfunction of the endoplasmic reticulum protein processing pathway. *Mol. Cell. Proteomics* (2014) doi:10.1074/mcp.M114.038786.

6. Hirata, H. *et al.* DICKKOPF-4 activates the noncanonical c-Jun-NH2 kinase signaling pathway while inhibiting the Wnt-canonical pathway in human renal cell carcinoma. *Cancer* **117**, 1649–1660 (2011).

7. Zhai, W. *et al.* High expression of the secreted protein dickkopf homolog 4: Roles in invasion and metastasis of renal cell carcinoma and its association with von Hippel-Lindau gene. *Int. J. Mol. Med.*

33, 1319–1326 (2014).

8. Fatima, S. *et al.* Dickkopf 4 (DKK4) acts on Wnt/B-catenin pathway by influencing B-catenin in hepatocellular carcinoma. *Oncogene* **31**, 4233–4244 (2012).

9. Liao, C. H. *et al.* Dickkopf 4 positively regulated by the thyroid hormone receptor suppresses cell invasion in human hepatoma cells. *Hepatology* **55**, 910–920 (2012).

10. Chi, H. C. *et al.* Thyroid hormone receptor inhibits hepatoma cell migration through transcriptional activation of Dickkopf 4. *Biochem. Biophys. Res. Commun.* **439**, 60–65 (2013).

11. Chouhan, S. *et al.* Glucose induced activation of canonical Wnt signaling pathway in hepatocellular carcinoma is regulated by DKK4. *Sci. Rep.* **6**, 27558 (2016).

12. Suresh, S. *et al.* SRC-2-mediated coactivation of anti-tumorigenic target genes suppresses MYC-induced liver cancer. *PLoS Genet.* (2017) doi:10.1371/journal.pgen.1006650.

13. Matsui, A. *et al. DICKKOPF-4* and *-2* genes are upregulated in human colorectal cancer. *Cancer Sci.* **100**, 1923–1930 (2009).

14. Tsukui, Y. *et al.* Dickkopf-4 gene expression is associated with differentiation and lymph node metastasis in colorectal cancer. *JGH Open* (2019) doi:10.1002/jgh3.12177.

15. He, S., Shen, J., Hu, N., Xu, X. & Li, J. DKK4 enhances resistance to chemotherapeutics 5-Fu and YN968D1 in colorectal cancer cells. *Oncol. Lett.* **13**, 587–592 (2017).

16. Pendás-Franco, N. *et al.* DICKKOPF-4 is induced by TCF/β-catenin and upregulated in human colon cancer, promotes tumour cell invasion and angiogenesis and is repressed by 1α,25-dihydroxyvitamin D3. *Oncogene* **27**, 4467–4477 (2008).

17. Chen, H. J. *et al.* Comprehensive models of human primary and metastatic colorectal tumors in immunodeficient and immunocompetent mice by chemokine targeting. *Nat. Biotechnol.* (2015) doi:10.1038/nbt.3239.

18. Baehs, S. *et al.* Dickkopf-4 is frequently down-regulated and inhibits growth of colorectal cancer cells. *Cancer Lett.* **276**, 152–159 (2009).

19. Wang, M. *et al.* Aberrant accumulation of Dickkopf 4 promotes tumor progression via forming the immune suppressive microenvironment in gastrointestinal stromal tumor. *Cancer Med.* (2019) doi:10.1002/cam4.2437.

20. Zeng, S. *et al.* Wnt/β-catenin signaling contributes to tumor malignancy and is targetable in gastrointestinal stromal tumor. *Mol. Cancer Ther.* (2017) doi:10.1158/1535-7163.MCT-17-0139.

21. Yang, X., Liu, Y., Li, W., Li, A. & Sun, Q. DKK4-knockdown enhances chemosensitivity of A549/DTX cells to docetaxel. *Acta Biochim. Biophys. Sin. (Shanghai).* (2017) doi:10.1093/abbs/gmx086.

22. Bahl, C., Singh, N., Behera, D. & Sharma, S. Genetic Variants in the Wingless Antagonist Genes (sFRP, DKK, and Axin2) Predict the Overall Survival and Prognosis of North Indian Lung Cancer Patients Treated with Platinum-Based Doublet Chemotherapy. *Cancer Biother. Radiopharm.* (2018) doi:10.1089/cbr.2018.2491.
